# Self-Assembly of Accumulated Sphingolipids into Cytotoxic Fibrils in Globoid Cell Leukodystrophy and Their Inhibition by Small Molecules In Vitro

**DOI:** 10.1021/acsnano.5c05498

**Published:** 2025-07-02

**Authors:** Sourav Kumar, Evelina Nikelshparg, Jana Pilátová, Ashim Paul, Vijay Kumar, Gil Koren, Roy Beck, Henrik H. Jensen, Daniel Segal

**Affiliations:** † Shmunis School of Biomedicine and Cancer Research, George Wise Faculty of Life Sciences, 26745 Tel Aviv University, Tel Aviv 6997801, Israel; ‡ Department of Life Sciences, Ben-Gurion University of the Negev, Beer Sheva 8855630, Israel; § Institute of Physics, Faculty of Mathematics and Physics, 563252 Charles University, Prague 2 121 16, Czech Republic; ∥ Molecular Foundry, Lawrence Berkeley National Laboratory, Berkeley, California 94720, United States; ⊥ Structural Biology & Bioinformatics Division, Indian Institute of Chemical Biology, Raja S. C. Mullick Road, Jadavpur, Kolkata 700032, India; # The Raymond & Beverly Sackler School of Physics and Astronomy, The Center for Nanoscience and Nanotechnology, and The Center for Physics and Chemistry of Living Systems, Tel Aviv University, Tel Aviv 6997801, Israel; ¶ Department of Chemistry, 1006 Aarhus University, Aarhus C 8000, Denmark; ∇ Sagol School of Neuroscience, Tel Aviv University, Tel Aviv 6997801, Israel

**Keywords:** globoid cell leukodystrophy, sphingolipids, self-assembly, galactosylceramide, galactosylsphingosine, small molecule inhibitors, apoptosis

## Abstract

Globoid cell leukodystrophy (GLD) is a rare hereditary inborn error of metabolism due to recessive mutations that cause loss of function of the enzyme galactosylceramidase (GALC). This results in the accumulation of the sphingolipids galactosylceramide (GalCer) and galactosylsphingosine (GalSph) in the lysosomes of neuronal cells. The accumulated GalCer and GalSph in cerebral macrophages of GLD patients are neurotoxic to oligodendrocytes and Schwann cells, leading to demyelination in the nervous system. The disease typically presents with infantile onset in the first six months of life and death by age 2. Here, we identified a supramolecular structure of GalCer and GalSph that may contribute to GLD pathology. Using biophysical assays commonly used for studying proteinaceous amyloids, e.g., amyloid-specific dyes, microscopical imaging, and a series of analytical methods (FTIR, PXRD, and SAXS), we demonstrate that both GalCer and GalSph can self-assemble in vitro into highly organized fibrils reminiscent of fibrils of amyloidogenic proteins. These fibrils exhibit significant cytotoxicity to both neuronal and oligodendroglial cells. Using an inhibitor of the GALC enzyme in cell culture to mimic the GLD pathophysiology, we could detect the accumulation of these fibrils in cells. We also observed that small molecules, which are bona fide inhibitors of proteinaceous amyloids, effectively mitigated the formation of the GalCer and GalSph fibrillar structures in vitro. Finally, the small molecule ameliorated the cytotoxic effects of the sphingolipid fibrils in SH-SY5Y cells, suggesting a potential avenue for therapeutic intervention in GLD orphan disease.

## Graphical abstract

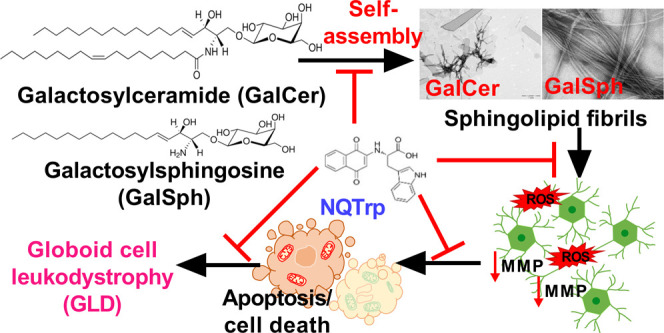

## Introduction

Lysosomal storage diseases (LSDs) are rare genetic disorders that result from mutations in genes associated with lysosomal functions. Despite their rarity, LSDs collectively affect 1 in 5,000 to 1 in 10,000 individuals.^1^ These mutations disrupt lysosomal enzymes, causing the accumulation of specific lipids and glycoproteins within lysosomes and, subsequently, leading to cellular dysfunction. Symptoms of LSDs vary widely and may include developmental delays, movement disorders, seizures, dementia, deafness, and blindness depending on the specific disorder and factors such as age at onset. Treatments such as enzyme replacement therapy, bone marrow transplantation, and more recently, gene therapy, have shown efficacy for some LSDs.^2^ However, the majority of LSDs currently lack effective treatments, posing ongoing challenges in clinical management and care.^3,4^ Sphingolipidoses constitute a significant class of LSDs, in which genetic mutations affect enzymes responsible for sphingolipid metabolism.^5^ Sphingolipids are part of a complex network of lipid molecules involved in various cellular processes, including cell membrane structure, signal transduction, and apoptosis.^6^ The main members of this subclass of diseases alongside the primary accumulating sphingolipids in them are Gaucher disease (GD)—glucosylceramide (GlcCer) and glucosylsphingosine (GlcSph); Fabry disease—globotriaosylceramide (Gb3); Niemann–Pick disease—sphingomyelin, sphingosine, glycosphingolipids, and cholesterol; Tay–Sachs disease—GM2 ganglioside; and metachromatic leukodystrophy—sulfogalactosylceramide.^7^

An additional sphingolipidosis is globoid cell leukodystrophy (GLD, also termed Krabbe disease). It is caused by the genetic loss of function of the lysosomal hydrolase galactosylceramidase (GALC) resulting in the accumulation of galactosylceramide (GalCer) and galactosylsphingosine (GalSph or psychosine) in myelinating (or Schwann) cells and in neurons of the central and peripheral nervous systems.^8^ More than 70 mutations distributed throughout the sequence of the *GALC* gene have been associated with severe clinical symptoms.^9^ The accumulation of GalCer and GalSph initiates a cascade of events, causing persistent deterioration of white matter tracts and neurodegeneration.^10^ GLD typically presents with infantile onset in the first six months of life; patients show a rapid and progressive course characterized by irritability, hypersensitivity to external stimuli, severe mental and motor deterioration, and death by 2 years of age.^11,12^ However, late-onset and adult forms of the disease have also been recognized.^13^ GLD affects 1 in 100,000 to 250,000 live births, but in some populations, its prevalence can be as high as 1 in 100 to 150 live births.^14^ The pathophysiological mechanism underlining GLD remains elusive. The existing evidence implicates those dysfunctional lysosomes in various LSDs, impairing autophagy, causing the buildup of protein aggregates and damaged organelles, as in more common neurodegenerative diseases.^15–18^ No therapeutics is currently available for GLD.^19^ Active experimental gene and cell therapy approaches for GLD are promising but require long-term monitoring to assess their efficacy and safety. Thus, drug development for GLD is an unmet need.

We have recently reported that GlcCer, which accumulates in GD, self-assembles in vitro into highly ordered fibrils resembling proteinaceous amyloids, such as amyloid-β and α-synuclein that are detectable using biophysical techniques commonly employed for amyloid aggregates.^20^ We further demonstrated that the GlcCer fibrils are cytotoxic.^20^ We further reported that small molecules, which are bona fide inhibitors of amyloid fibril formation, can mitigate GlcCer fibrils, suggesting a possible therapeutic approach by targeting the self-assembly of the accumulating metabolite.^20^ In this study, we investigated the formation and structure of GalSph and GalCer fibrils in vitro using a complementary series of biophysical assays, including Thioflavin S (ThS) binding, turbidity assay, electron microscopy, fluorescence microscopy, and FTIR and Raman spectroscopy. Based on X-ray scattering analysis of the GalCer and GalSph aggregates, we propose two plausible structural models: twisted-ribbon- and straight filament-like morphology, respectively. We further demonstrate in vivo aggregates of lipid in SHSY-5Y neuroblastoma cells. GalCer and GalSph fibrils showed cytotoxicity by inducing cellular apoptosis and impairment of mitochondrial integrity, suggesting a possible pathogenic mechanism in GLD. Finally, we demonstrate the effective mitigation of GalCer and GalSph aggregation in vitro by several small molecules that are bona fide inhibitors of proteinaceous amyloids.

## Results

### GalCer and GalSph Self-Assemble In Vitro Like Proteinaceous Amyloids

Two sphingolipid metabolites, GalCer and GalSph, accumulate in the GLD and trigger pathogenesis. Given the report that glucosylceramide (GlcCer), which accumulates in Gaucher disease (GD), can self-assemble in vitro to form fibrils with amyloid-like characteristics,^20^ we examined here whether these attributes extend to the sphingolipids associated with GLD. To that end, we dissolved GalCer and GalSph (Figure S1) powders in 100% DMSO to make stock solutions (10 mM), which were further diluted before use with either PBS (pH 7.4), acetate buffer (hereafter AB, pH 4.5), or DDW (pH 6) to make various concentrations. The kinetics of the self-assembly of GalCer and GalSph was monitored using the Thioflavin S (ThS) binding assay. ThS is a commonly used amyloid probe, similar to Thioflavin T, which allows quantification of amyloids formed in vitro. Monomeric GalCer (Figure 1a,b) and GalSph (Figure 1c,d) were incubated at different concentrations (10–300 μM) in PBS (pH 7.4) or at AB (pH 4.5), which matches the pH of the lysosome where these sphingolipids accumulate, with 30 s of shaking at 37 °C. The curve showing enhancement of ThS fluorescence intensity over time (Figure 1a–d) suggests a pattern similar to the reported self-assembly of proteinaceous amyloids and GlcCer.^20^ Furthermore, there was a clear concentration-dependent increase in ThS fluorescence intensity, suggesting an enhanced rate of aggregation of sphingolipids at higher concentrations. The results in Figure 1 indicate that the kinetics of aggregation of the sphingolipids were similar, but GalSph aggregated more rapidly than GalCer. Additionally, the end point ThS fluorescence intensity of GalSph (Figure S2b) was >5-fold higher than that of GalCer (Figure S2a). Subsequent analyses of the structure of the resultant fibrils may bear this difference.

**Figure 1:**
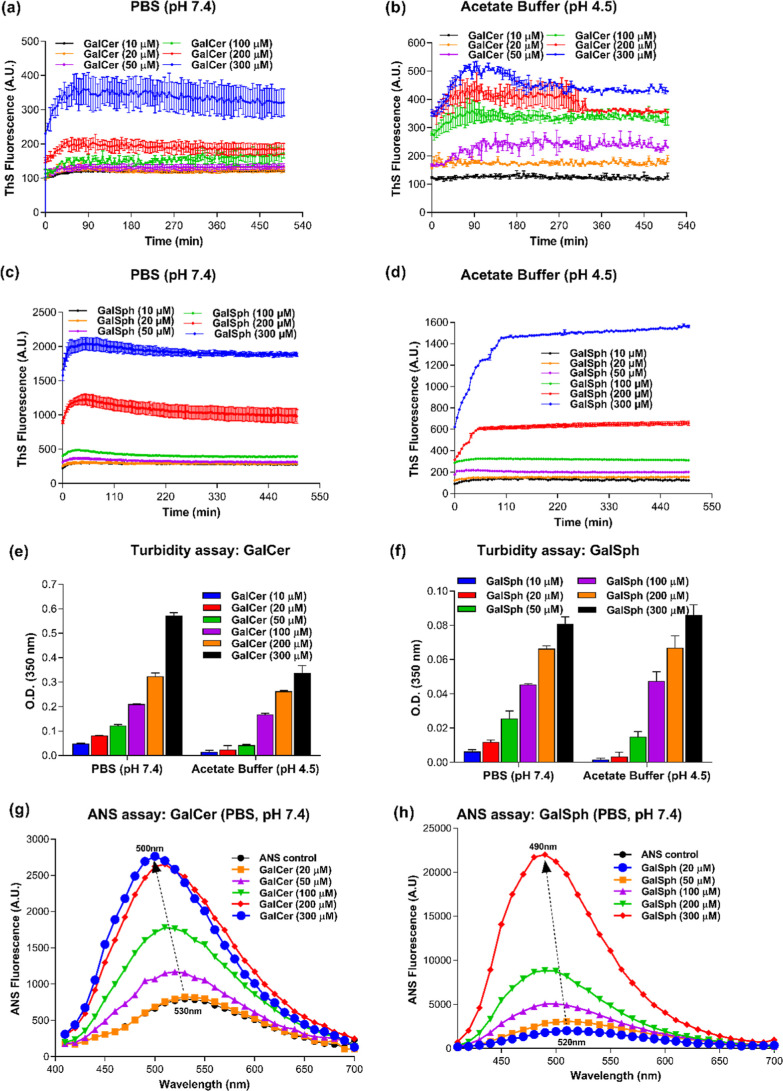
Self-assemblies of GalCer and GalSph into amyloid-like fibrils in vitro. Time-dependent ThS fluorescence at 485 nm for the aggregation of GalCer at different concentrations in (a) PBS pH 7.4 and (b) AB pH 4.5 and GalSph in (c) PBS pH 7.4 and (d) AB pH 4.5. Turbidity assay of (e) GalCer and (f) GalSph in PBS pH 7.4 and AB pH 4.5 at different concentrations. ANS-binding assay for the aggregation of (g) GalCer and (h) GalSph at different concentrations (20–300 μM). ANS emission spectra of GalCer and GalSph were recorded at the wavelength range of 400–700 nm. A sharp blue shift was observed for both of the sphingolipids (indicated by a broken arrow). The values represent mean ± SEM, *n* = 3 from independent experiments.

An independent measure of the kinetics of GalCer and GalSph aggregation was assessed by a turbidity assay. Monomeric GalCer (Figure 1e) and GalSph (Figure 1f) were dissolved either in PBS (pH 7.4) or AB (pH 4.5) at 37 °C for 24 h. Absorbance was recorded at 0 h and after 24 h of incubation. At both conditions examined, the solutions of the two sphingolipids became gradually turbid over the incubation period very similar to the increasing turbidity reported for amyloidogenic proteins^21,22^ and GlcCer.^20^ At a higher concentration (300 μM), the solutions became more turbid than at lower concentrations, evident by the presence of white precipitate inside the wells (Figure S3a,b).

To validate the amyloid-like characteristics of the GalCer and GalSph aggregates, we used the dye 1-anilinonaphthalene-8-sulfonic acid (ANS), which is commonly employed to identify the hydrophobic pockets of amyloids by a blue shift in its emission maximum upon binding to protein aggregates, as well as to self-assemblies of GlcCer.^20^ A sharp blue shift from ∼530 to ∼500 nm for GalCer and from ∼530 to ∼490 nm for GalSph with enhanced intensity was observed when ANS bound to different concentrations of these sphingolipids, whereas ANS alone (control) exhibited emission solely at ∼530 nm (Figure 1g,h). This indicates an amyloid-like nature of the GalCer and GalSph aggregates.

Congo red (CR) is an amyloid-detecting dye, which upon binding to the cross-β sheets of amyloids exhibits golden apple-green birefringence under cross-polarized light.^23^ GalCer and GalSph aggregates were taken from the 200 μM samples at the end of the turbidity assay (Figure 2a­(i,ii)) and stained with CR for 15 min. The aggregates of both sphingolipids exhibited typical amyloid-specific apple-green birefringence. Likewise, staining of the self-assemblies with ThS revealed a green color under a fluorescent microscope (Figure 2b­(i,ii,iii)) in line with their amyloid-like characteristics as revealed by ANS and CR binding.

**Figure 2:**
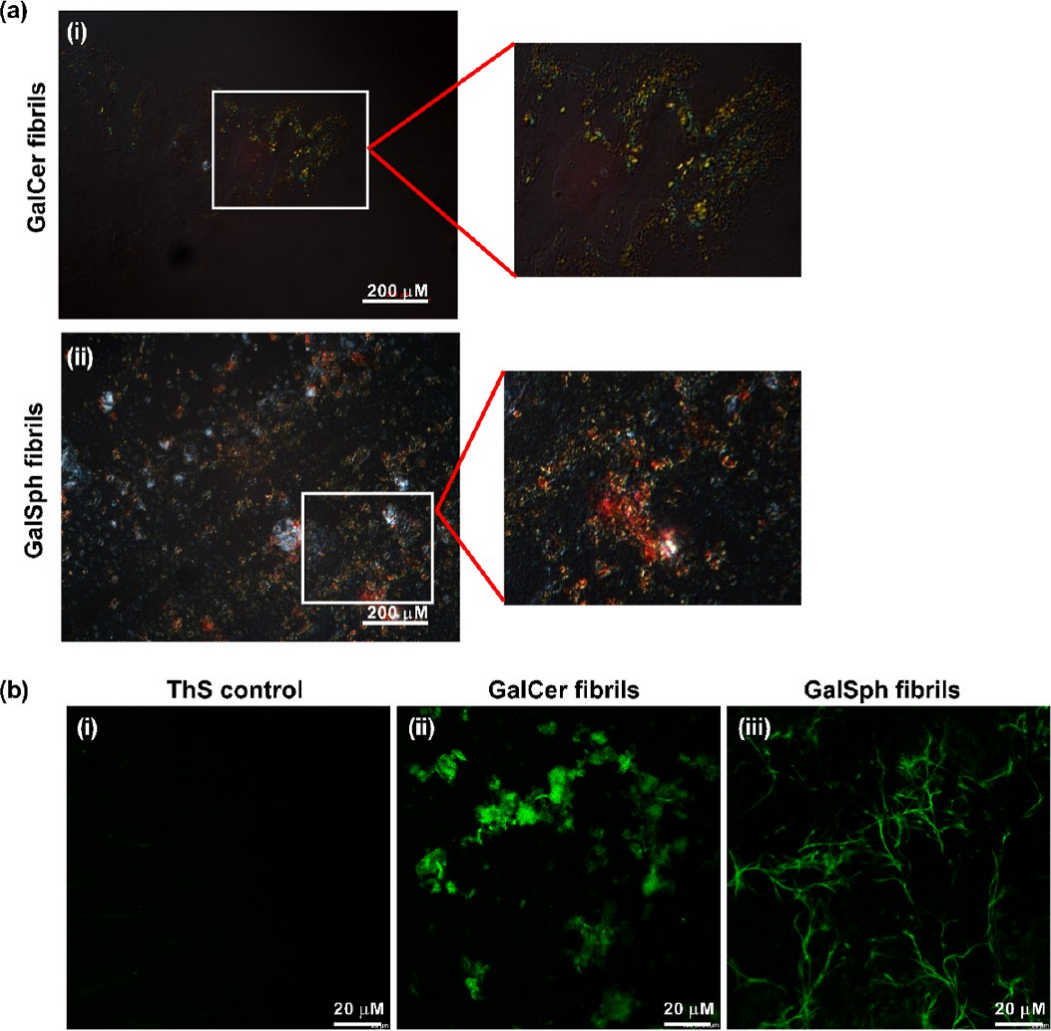
Microscopic images of GalCer and GalSph self-assemblies in vitro. (a) Congo red birefringence of (i) GalCer and (ii) GalSph fibrils after staining with the Congo red dye under a polarization microscope. Enlarged views of the images shown as emerging from (i) and (ii) are the cropped areas and are color-corrected for better visualization of CR birefringence. (b) Confocal microscopic imaging of (i) ThS control, (ii) GalCer, and (iii) GalSph upon staining with ThS dye. Control contained ThS + PBS only.

We next examined the morphology of the self-assemblies of GalCer and GalSph at the end point of the ThS kinetics assay, using TEM. Highly ordered fibrillary structures were observed (Figure 3a,b), very reminiscent of fibrils reported for proteinaceous amyloids and for the self-assemblies of GlcCer.^20^ After 24 h incubation in PBS (pH 7.4), the GalCer (Figure 3a­(i,ii)) fibrils appeared as twisted ribbons and flat ribbon-like structures with an average diameter of 40 ± 14 (Figure 3c­(i)) nm and average pitch of 217 ± 45 (Figure 3c­(ii)), comparable to the previously reported helical ribbon-like structure of GlcCer.^20^

**Figure 3:**
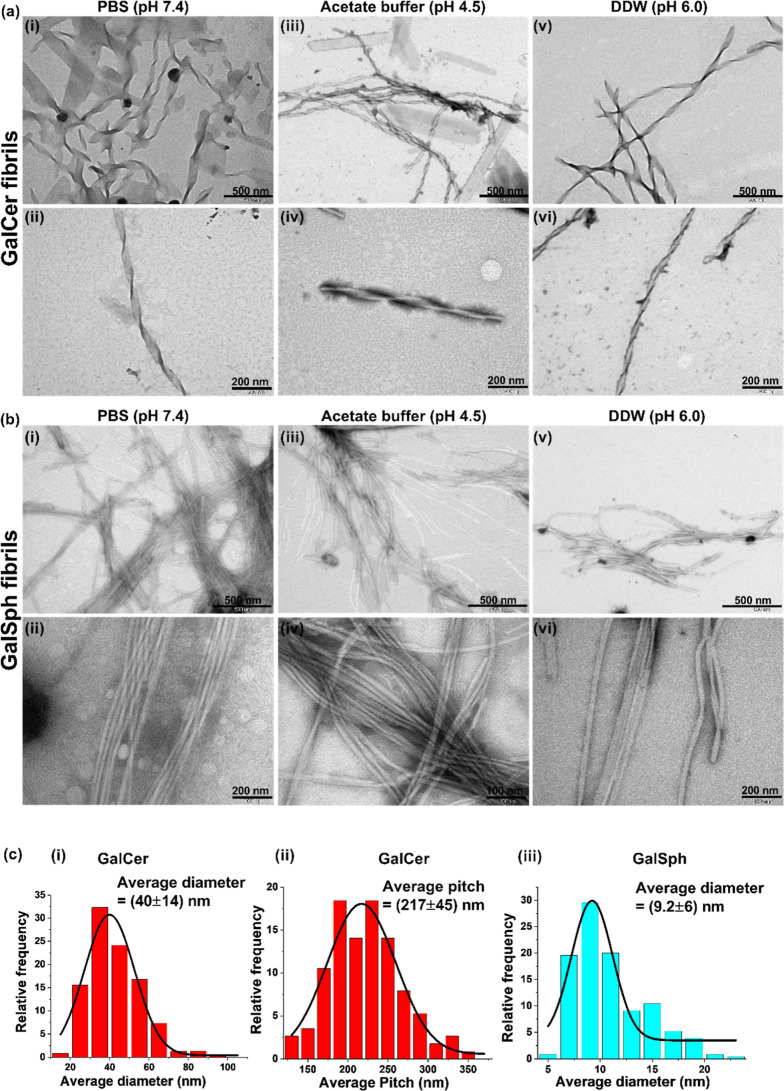
Transmission electron microscopy (TEM) imaging of GalCer and GalSph fibrils. (a) The TEM micrographs show morphology of GalCer fibrils in (i,ii) PBS pH 7.4, (iii,iv) AB pH 4.5, and (v,vi) DDW pH 6.0. (b) GalSph fibrils in (i,ii) PBS pH 7.4, (iii,iv) AB pH 4.5, and (v,vi) DDW pH 6.0. (c) Average diameter of GalCer (i) and GalSph (iii) and the average pitch of GalCer (ii) were calculated from TEM images using ImageJ. Calculations are based on 230 and 110 different regions of the fibrils of GalCer and GalSph, respectively.

In contrast, following 24 h incubation at PBS (pH 7.4), the GalSph (Figure 3b­(i,ii)) self-assemblies appeared as bundles of straight fibrillar networks comparable to typical proteinaceous amyloid (e.g., amyloid-β or α-synuclein).^24–29^ The average diameter of the GalSph fibrils at pH 7.4 was 9.2 ± 6 nm (Figure 3c­(iii)). These morphologies of GalCer (Figure 3a­(iii,iv)) and GalSph (Figure 3b­(iii,iv)) were maintained also at acidic pH 4.5. The fibrils formed by GalCer (Figure 3a­(v,vi)) and GalSph (Figure 3b­(v,vi)) in an aqueous environment (DDW, pH 6.0) retained structures similar to those reported for GlcCer fibrils.

We also determined how soon the fibrillar structures appeared after the initiation of the aggregation process. To that end, GalCer and GalSph samples were taken at time 0 of the aggregation experiment, i.e., when the sphingolipid stock solutions in DMSO were added to the PBS buffer, as well as at 30 min, 60 min, 3 h, 6 h, and 12 h of aggregation and examined them by TEM. As shown in Figures S4 and S5, samples of all time points before 12 h showed no detectable mature fibrils.

These fibrils remained very stable, even after incubation at 37 °C in PBS (pH 7.4) for 30 and 60 days (Figure S6). TEM analysis also showed that the fibrils maintained their morphology under a range of temperatures (25–80 °C) indicating thermal stability (Figure S7) as reported for GlcCer fibrils.^20^

### Structural Details of the GalCer and GalSph Assemblies

The above-mentioned results of ThS, turbidity, and TEM analyses collectively suggest the self-assembly of GalCer and GalSph. Therefore, we proceeded to conduct a detailed structural characterization of these fibrils in DDW (pH 6.0) to avoid any possible interference from the salts in PBS.

We first calculated the diameter and pitch of the fibrils as the average of their values measured for 50 different fibrils generated in PBS (pH 7.4) using the ImageJ software. TEM images indicated a twisted-ribbon-like fibrillar structure of GalCer fibrils with an average diameter of 40 ± 14 nm and twist (pitch) of 217 ± 45 nm (Figure 3c­(i,ii)). These parameters resemble those previously reported for the twisted helical GlcCer fibrils (Avg. pitch 184 ± 18 nm and 52 ± 11 nm in diameter).^20^ In contrast, the GalSph (Figure 3c­(iii)) fibrils, which under TEM displayed a straight filament-like morphology without twists, have a considerably smaller average diameter of 9.2 ± 6 nm. A possible explanation for the difference in the fibril structure between GalSph and GalCer and GlcCer will be alluded to in the discussion.

Additional structural insights into the sphingolipid fibrils were sought using Fourier-transform infrared (FTIR), Raman microspectroscopy, powder X-ray diffraction (PXRD), and small-angle X-ray scattering (SAXS) analyses. FTIR spectroscopy provides information about different functional groups present in a molecule and is widely used for characterizing the molecular interaction between lipids, including sphingolipids, within bilayers in aqueous media.^30–32^ FTIR spectroscopic analysis was carried out for the fibrils compared to their respective monomers (Figure 4d).

**Figure 4:**
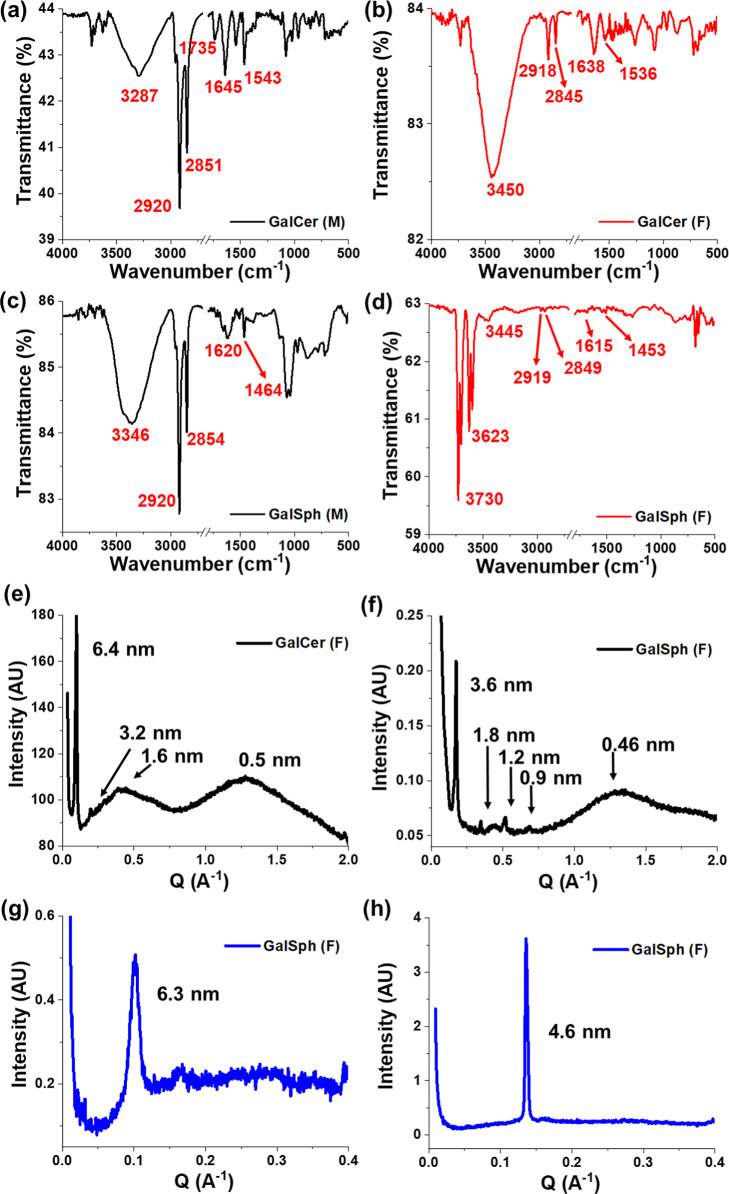
Structural parameters of the sphingolipid fibrils. FTIR spectra of the monomers of GalCer (a) and GalSph (c) and of fibrils of GalCer (b) and GalSph (d). PXRD of the fibrils of GalCer (e) and of GalSph (f). SAXS pattern of the fibrils of GalCer (g) and of GalSph (h).

The FTIR spectrum of the GalCer monomers exhibited two sharp peaks at 2851 cm^–1^ and 2920 cm^–1^, corresponding to the characteristic symmetric and asymmetric stretching vibration of the ceramide CH_2_ groups, respectively, indicating a fluid-like phase (Figure 4a). A sharp peak observed at 1543 cm^–1^, attributed to the stretching vibration of amide II groups, possibly arises due to the N–H bending vibration and C–N stretching vibration. Another peak observed at 1645 cm^–1^ corresponds to the stretching vibration of amide I groups, reflecting a fluid-like phase (Figures 4a and S8a). An additional broad peak detected at 3287 cm^–1^ represents a merge peak of the amide A and hydroxyl groups of the polar sugar unit of GalCer (Figure 4a). In contrast, the fibrils of GalCer exhibited two less-intense downshifted peaks at 2845 cm^–1^ and at 2918 cm^–1^, attributed to the ceramide CH_2_ groups with tight packing. Additional two less-intense broad peaks at 1638 cm^–1^ and at 1536 cm^–1^, corresponding to the amide I and amide II groups. These observations clearly indicate the presence of intermolecular hydrogen bonding between the amide group and hydroxyls of the hexose ring of the sugar, and fatty acid chain, and suggest a gel-like structure formation by GalCer (Figures 4b and S8b).^20,32^ An additional broad peak noted at 3450 cm^–1^ possibly represents the hydroxyl groups of the galactosyl headgroup and the NH of amide A of GalCer, which are hydrogen-bonded with the intermolecular hydroxyl group of the glycan unit or with the environmental H_2_O (Figure 4b). Similar to the GalCer monomers, GalSph monomers also exhibited two sharp peaks at 2854 cm^–1^ and 2920 cm^–1^, attributed to the characteristic symmetric and asymmetric stretching vibration of the lipidic CH_2_ groups, reflecting a lipidic fluid-like phase (Figure 4c). Additional two peaks observed at 1620 cm^–1^ and 1464 cm^–1^, corresponding to the N–H bending or H-bonded OH deformation and bending vibration of the CH_2_ groups, respectively, indicate the presence of free NH_2_ (Figures 4c and S8c). Another broad peak, observed at 3346 cm^–1^, reflects the O–H and N–H stretching vibrations of the primary amine. In contrast, the fibrils of GalSph exhibited lesser-intense and broader peaks near 2849 cm^–1^ and 2919 cm^–1^, attributed to the characteristic symmetric and asymmetric stretching vibration of the sphingosine CH_2_ groups, suggesting more packed and ordered CH_2_ groups in a gel-like aggregated form of the sphingolipid (Figure 4d). Another two less-intense downshifted broad peaks at 1615 cm^–1^ and 1453 cm^–1^ correspond to the N–H bending or H-bonded OH deformation and bending vibration of the CH_2_ groups, respectively. They suggest the presence of intermolecular hydrogen bonding between the primary amine and hydroxyls on the hexose ring of the sugar and sphingosine chain. This leads to the formation of H-bonded lamellae and gel-like structure (Figures 4d and S8d). In addition, three other peaks were observed at 3445 cm^–1^, 3623 cm^–1^, and 3730 cm^–1^, reflecting the stretching vibrations of the O–H and N–H groups, possibly due to the presence of a hydrogen-bonded hydroxyl group (of the sphingosine unit), with the surrounding water molecules or the galactose headgroup, and a free hydroxyl group.^20,32^

In addition to FTIR spectra, Raman microspectroscopy revealed other vibrational spectroscopic differences between the monomer and fibrillar GalCer and GalSph. Vibrations of acyl chains dominate the overall spectra of both GalCer and GalSph, with the amide band contributing to the ceramide spectrum and negligible vibrations of pyranose ring. All spectra are normalized to the intensities of the CH_2_ scissoring deformation peaks, i.e., 1438 cm^–1^ for GalCer and 1445 cm^–1^ for GalSph as well as the high-wavenumber region (2700–3000 cm^–1^) of CH stretching (Figure 5). These regions do not exhibit intensity changes due to signal polarization occurring in fibrillar forms. Due to structural organization, some covalent bonds oriented parallel to the polarization plane of the incident 532 nm laser beam show significant intensity enhancement. In our case, arbitrarily, the maximum signal polarization occurs at 90°, compared to the minimum at a perpendicular orientation of 0°. Subtraction of these signals yields differential spectra, showing a two- to multiple-fold enhancement of 1062 cm^–1^ (C–C asymmetric stretching), 1111 and 1130 cm^–1^ (C–C stretching), 1295 cm^–1^ (CH_2_ twisting), and 1670 cm^–1^ (C=C stretching), while there is no change in 1445 and 1460 cm^–1^ (CH deformations), and 1650 cm^–1^ (NH in-plane or amide I band), the latter being present only in GalCer. Conversely, this behavior implies that the fibrils have properties of a highly organized, crystalline-like structure, providing further independent spectroscopic confirmation of their structural organization. Simultaneously, awareness of this phenomenon, which explains the fluctuating signal intensities, is important for accurate data interpretation. Additionally, we provide the average spectrum as a reference, matching the published ceramide spectra^33–35^ for GalCer, and report Raman spectra of GalSph here. The difference between the GalCer monomer and fibril is evident in the absence of peaks at 677 and 711 cm^–1^ in the monomer, although these peaks have been reported in ceramide spectra^33–35^ without specifying the particular structural form. GalSph monomers have broader bands compared to the sharper peaks of fibrils with a less resolved vibrational signal and stronger CH stretching at high wavenumbers.

**Figure 5:**
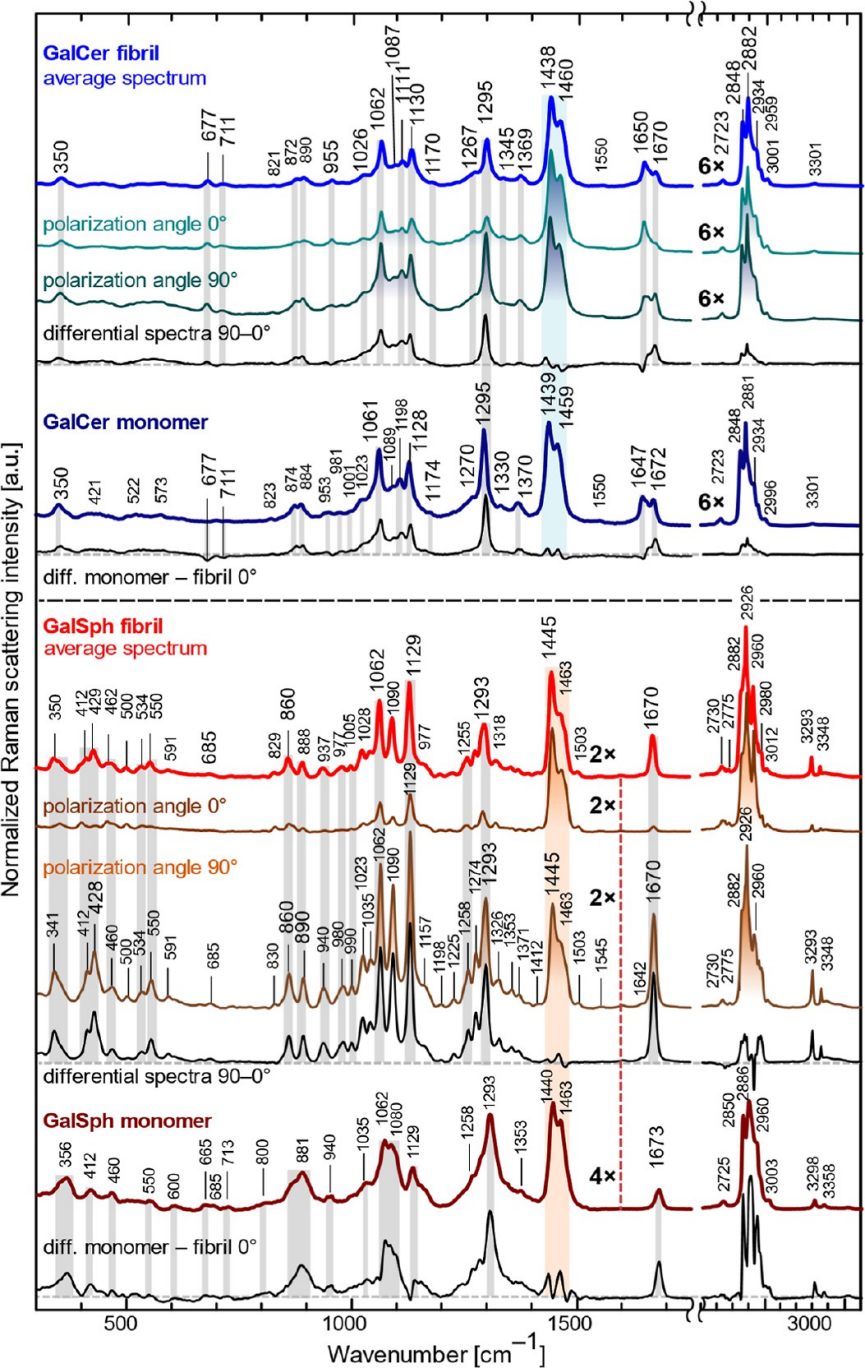
Raman microspectroscopy of monomeric and fibrillar GalCer and GalSph. The spectra are normalized to the intensity at 1438 cm^–1^ for GalCer and at 1445 cm^–1^ for GalSph, highlighted in blue and red semitransparent rectangles, respectively. The figure shows the dependence of the Raman signal for fibrillar forms oriented at 0° and 90°. The average spectrum is the mean of the Raman signal polarization at the extreme angles of 0° and 90°. Differential spectra refer to the subtraction of the signals at two limiting angles, 90° and 0°, and a comparison of the monomeric and fibrillar forms oriented at 0° (diff. monomer–fibril 0°). The intensity scale of CH vibrations at high wavenumbers has been reduced by factors of 2×, 4×, or 6×, proportionally to the rest of the spectra.

PXRD is a useful tool to investigate the interactions within the self-assembled sphingolipid molecules and to understand the molecular arrangement for the formation of their lateral domains in the lipid bilayer.^20,36^ PXRD spectra of the GalCer and GalSph fibrils exhibited mainly one pronounced peak with the diffraction wave vector *Q* = 0.098 Å^–1^ and 0.174 Å^–1^, respectively, corresponding to the *d*-spacing of 6.4 and 3.6 nm, respectively (Figure 4e,f). Additionally, we observed less-intense second and third harmonic peaks along with the pronounced peak, indicating a gel phase formation, possibly suggesting a liquid crystalline nature of the sphingolipid fibrils, as reported for GlcCer fibrils.^20^ Additionally, the GalCer, and GalSph fibrils exhibited a broad peak at ∼0.5 nm, and ∼0.46 nm, respectively (Figure 4e,f), a feature of well-ordered chain packing, indicating the presence of a gel phase, characteristic of reported lipid aggregates.^20,37–39^

Lastly, to gain further molecular level information regarding the sphingolipid fibrils and to validate the PXRD results, we performed SAXS measurements. We observed that the GalCer and GalSph fibrils exhibited a single reflection in the low-angle region (SAXS) with a scattering wave vector (*Q*) of 0.1 Å^–1^ and 0.136 Å^–1^, respectively. These wave vectors correspond to correlation lengths (*d* = 2π/*Q*) of 6.3 and 4.6 nm, respectively (Figure 4g,h). We suggest that these correlation lengths indicate the repeat distance of a lamellar phase formed by the lipid molecules and corroborate the PXRD and FTIR results.^19,40,43^

Collectively, these results suggest that the GalCer and GalSph fibrils are arranged to form a bilayer structure and amyloid-like fibrils in aqueous media very comparable to those reported for GlcCer.^20^

### Cytotoxicity of GalCer and GalSph Fibrils Involves Induction of Cellular Apoptosis and Disruption of Mitochondrial Integrity

GLD pathologies associated with GalCer and GalSph accumulations have been identified in various cell types: skin fibroblasts, macrophages, and glial and neuronal cells.^40^ Here, we examined the cytotoxicity of GalCer and GalSph fibrils compared to their monomeric forms toward neuronal (SH-SY5Y) and oligodendrocyte (DDR1) cells. For this purpose, first monomeric GalCer and GalSph were dissolved at different concentrations (1–200 μM) in DMEM/F12 or DMEM culture medium without cells for 24 h to generate sphingolipid fibrils that were confirmed by TEM (Figure S9). Next, the resultant fibrils or the corresponding sphingolipid monomers (stock solution prepared in 100% DMSO) were incubated with the cells for 24 h, after which cytotoxicity was monitored using an MTT cell viability assay. Cells untreated with GalCer or GalSph fibrils served as a control. A culture medium containing 1% DMSO was used as a vehicle control. A significant dose-dependent reduction in cell viability was observed after treatment with GalCer (Figure 6a,b) and GalSph (Figure 6c,d) fibrils, suggesting that the fibrils of both sphingolipids are cytotoxic to both SH-SY5Y and DDR1 cells, as reported for GlcCer fibrils.^20^ Interestingly, the GalSph fibrils were much more toxic than those of GalCer as evident from significantly greater reduction of viability which was attained at 200 μM for GalCer vs GalSph (Figure 6c,d). Cytotoxicity of the monomeric forms GalCer and GalSph was also examined. For this purpose, the cells were treated with different concentrations of monomeric GalCer and GalSph, and cell viability was monitored after 3, 6, and 12 h of incubation in SH-SY5Y cells. A significant reduction in cell viability was noted starting at 6 h of incubation with sphingolipids (Figure S10a–f). It could be due to monomeric sphingolipids turning into aggregated fibrils and showing cytotoxicity. These results are in line with the pathogenicity of the accumulated GalCer and GalSph in GLD.^41^

**Figure 6:**
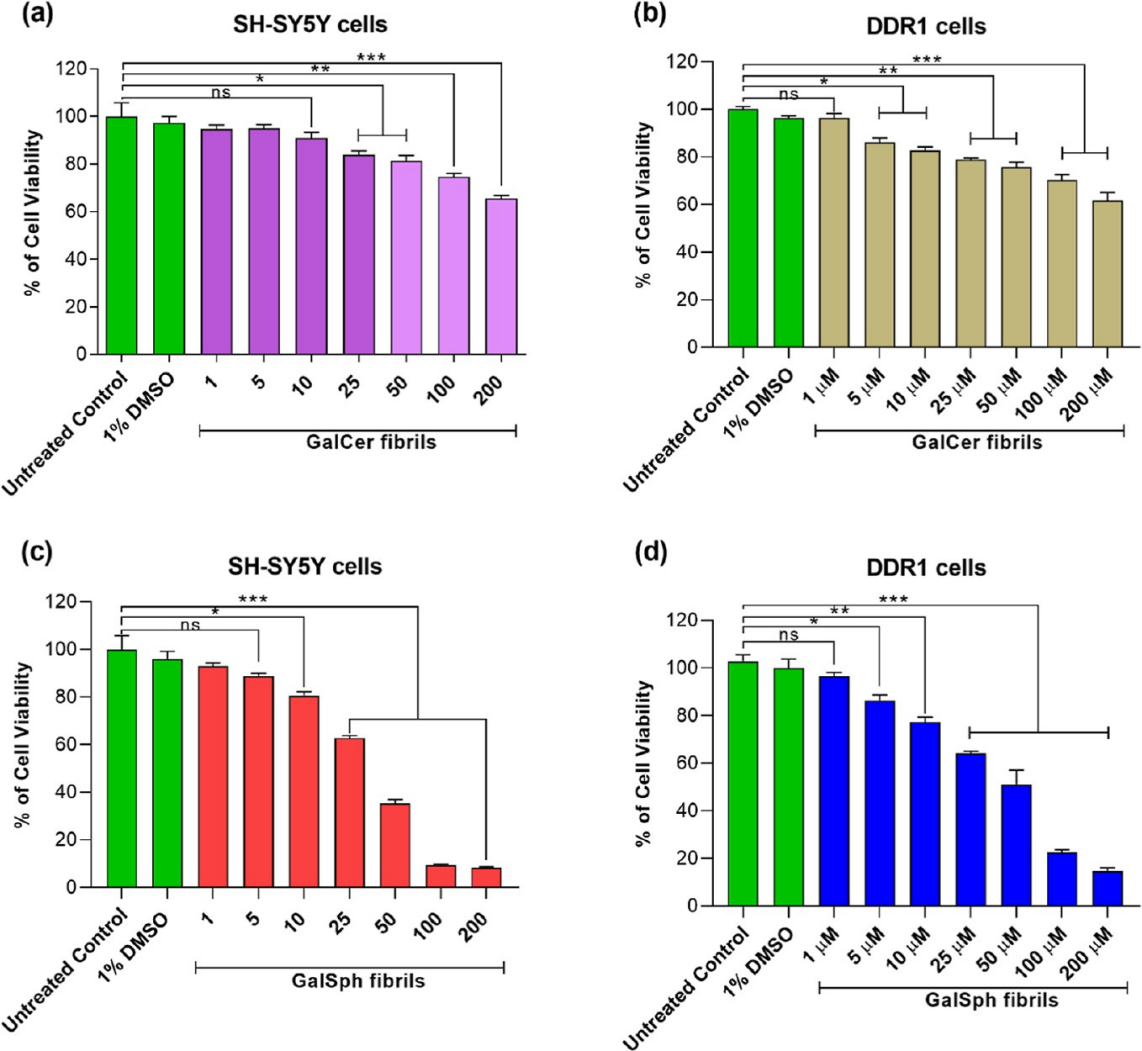
Cytotoxicity of GalCer and GalSph fibrils toward SH-SY5Y and DDR1 oligodendrocyte cells. Cells were treated with GalCer (a,b) and GalSph (c,d) fibrils for 24 h and cell viability was measured by MTT assay. Untreated control reflects the medium without GalCer or GalSph fibrils. A culture medium with 1% DMSO was used as a vehicle control. The data are represented as percentage cell viability. Each bar represented as mean ± SEM, *n* = 6. Statistical significance was analyzed using one-way ANOVA followed by the Tukey multiple comparison posthoc test, *p < 0.05, **p < 0.01, ***p < 0.001 vs untreated control.

To examine whether the cytotoxicity of the sphingolipid fibrils was due to apoptotic cell death, we used annexin V-FITC and propidium iodide (PI) labeling, followed by flow cytometric analysis. Externalization of phosphatidylserine from the lipid bilayer upon cell death is a hallmark indicator of apoptosis due to changes in the membrane asymmetry.^42,43^ Annexin V binds to phosphatidylserine when it is present in the outer leaflet of the plasma membrane. Based upon the results of the MTT assay, we chose for these experiments different concentrations of GalCer fibrils (100 μM and 200 μM) and of GalSph fibrils (10 μM, 25 μM, and 50 μM), prepared as described above, and used 1% DMSO as a vehicle control (Figure 7a–h). Flow cytometric analysis revealed that the percentage of cells in early apoptosis (annexin V-FITC-positive population) and late apoptosis (annexin V-FITC-positive/PI-positive population) increased markedly in a dose-dependent manner following treatment with GalCer fibrils (100 μM and 200 μM) (Figure 7c,d) or GalSph fibrils (10 μM, 25 μM, and 50 μM), respectively (Figure 7e–h), as compared to untreated control and vehicle control cells (Figure 7a,b). This indicates late apoptosis induced by the GalSph fibrils.

**Figure 7:**
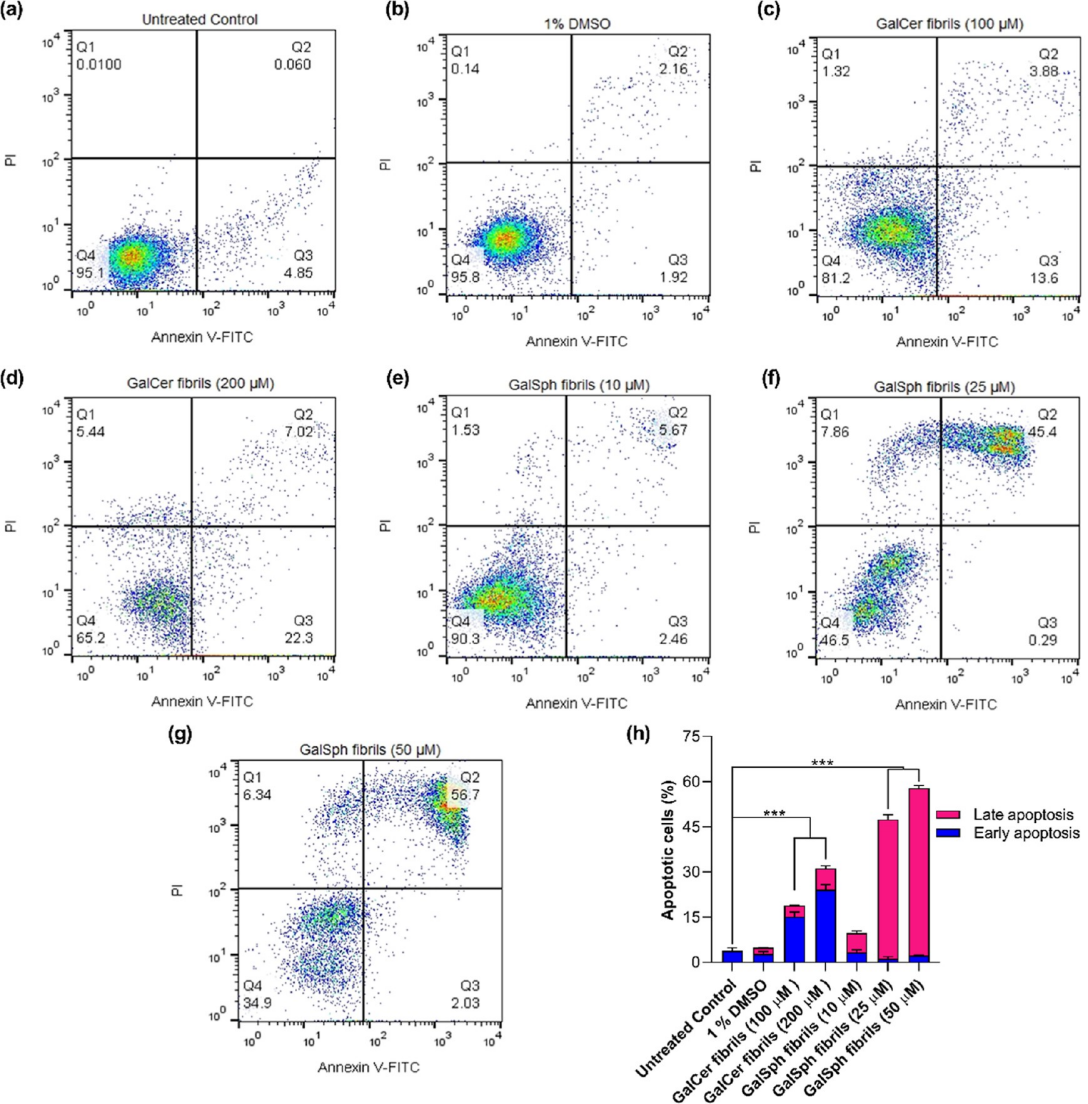
Flow cytometric analysis of apoptotic activity in SH-SY5Y cells using the Annexin V-FITC/PI assay. Cells were treated with GalCer fibrils (100 μM and 200 μM) and GalSph fibrils (10 μM–50 μM) for 24 h at 37 °C. After incubation, annexin V-FITC and PI were added, and apoptosis was assessed by flow cytometry using a single laser emitting excitation light at 488 nm. The scatter plots represent apoptosis analysis in (a) untreated control, (b) 1% DMSO vehicle control, (c,d) GalCer-treated cells, and (e–g) GalSph fibril-treated cells. The quadrants indicate the following: Q1—PI (+) (necrotic cells), Q2—Annexin V-FITC (+) PI (+) (late apoptotic cells), Q3—Annexin V-FITC (+) PI (−) (early apoptotic cells), and Q4—Annexin V-FITC (−) PI (−) (live cells). (h) The bar graph shows the percentage of early and late apoptotic cells in GalCer- and GalSph-treated conditions. Data are presented as mean ± SEM, *n* = 3. Statistical significance was analyzed using one-way ANOVA followed by Tukey’s multiple comparison posthoc test, ***p < 0.001 vs untreated control.

A unique feature of the early stages of apoptosis is the disruption of active mitochondrial function evident as changes in the membrane potential and alterations of the oxidation–reduction potential of the mitochondria.^44^ We examined whether the apoptosis induced by the sphingolipid fibrils was associated with modification of the mitochondrial membrane potential (MMP) using JC-1 to detect the changes in mitochondrial integrity in terms of potential difference. JC-1 is a membrane-permeable dye that exhibits potential-dependent accumulation in mitochondria. A decrease in its red/green ratio of fluorescence intensity signifies a reduction in mitochondrial integrity.^45^ Incubation of the SH-SY5Y cells with the GalCer (Figure 8b,c) and GalSph (Figure 8d–f) fibrils markedly increased the percentage of cells of JC-1 monomers exhibiting green fluorescence in a dose-dependent manner relative to the control cells that were not treated with either fibrils (Figure 8g). The maximal change in the percentage of cells of JC-1 monomers displaying green fluorescence was observed when the cells were treated with 50 μM GalSph fibrils (Figure 8f) or 200 μM GalCer fibrils (Figure 8c) compared to the untreated control cells. These results are in line with those of the cytotoxicity assay (Figure 6a–d) which indicated a greater effect of GalSph than GalCer and suggest that this cytotoxicity may be mediated by alteration of the MMP following induction of the apoptotic machinery.

**Figure 8:**
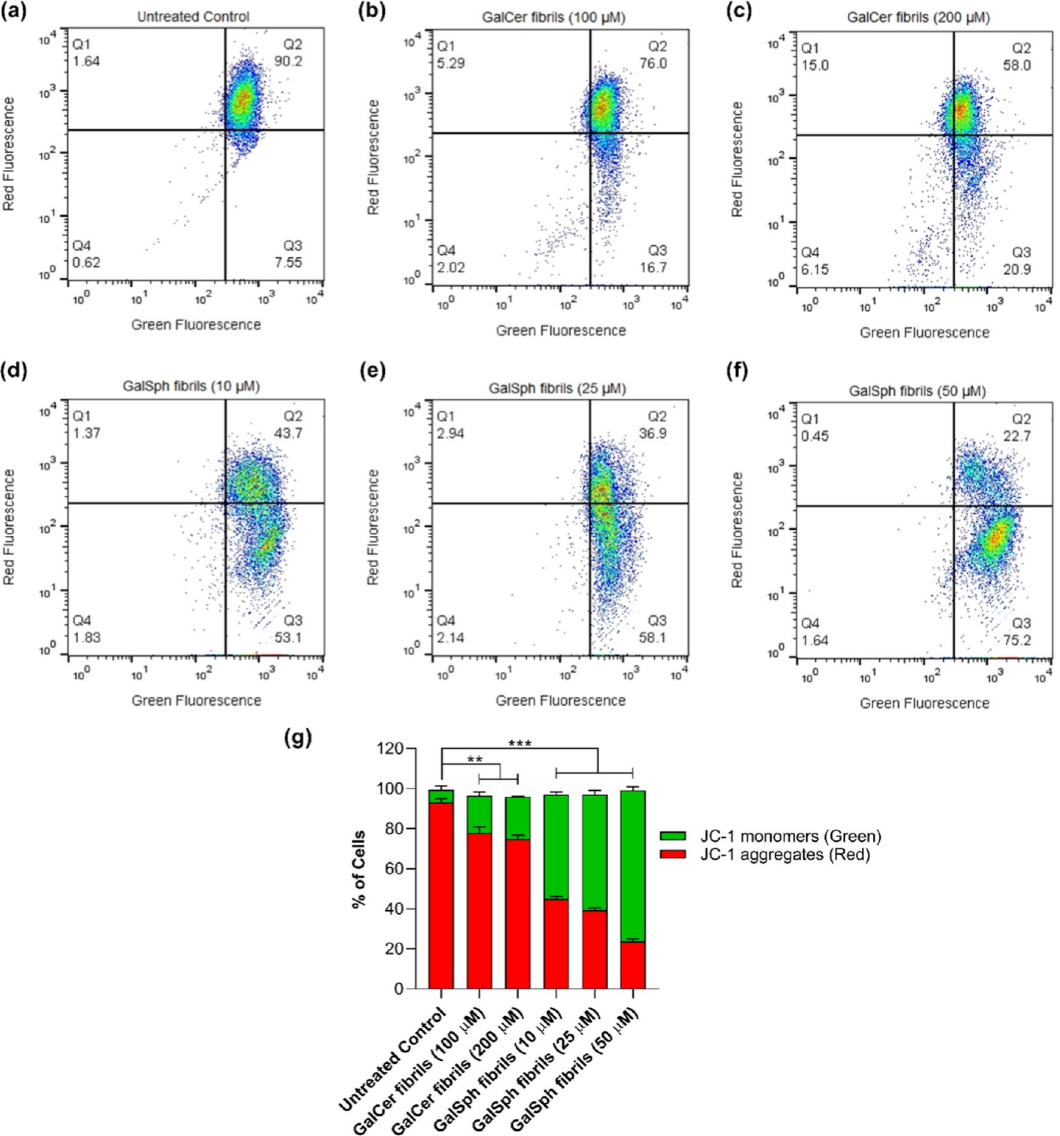
Flow cytometric analysis of mitochondrial membrane potential in SH-SY5Y cells using JC-1 dye. Flow cytometry analysis of loss of MMP was monitored by JC-1 dye. Cells were treated with GalCer and GalSph fibrils for 24 h, as described in the apoptosis assay. The scatter plot represents JC-1 red fluorescence (aggregates) versus JC-1 green fluorescence (monomers), indicating mitochondrial membrane potential. A shift toward increased green fluorescence signifies mitochondrial depolarization. The plots correspond to (a) untreated control and (b) cells treated with 100 μM GalCer fibrils, (c) 200 μM GalCer fibrils, (d) 10 μM GalSph fibrils, (e) 25 μM GalSph fibrils, and (f) 50 μM GalSph fibrils. (g) The bar graph represents the quantification of JC-1 monomer-to-aggregate in GalCer- and GalSph-treated cells, expressed as the percentage of cells in each condition. Values are mean ± SEM, *n* = 3. Statistical significance was analyzed using one-way ANOVA followed by the Tukey multiple comparison posthoc test, **p < 0.01, ***p < 0.001 vs untreated control.

Cytotoxicity is often reflected by oxidative stress. We attempted to evaluate the mitochondrial redox state in the fibril-treated cells by examining the level of reduced cytochromes in them. This was achieved using Raman microspectroscopy analysis of the living cells. Treatment with GalSph fibrils results in a decrease in the main peaks of reduced (Fe^2+^) cytochromes: 747 and 1313 (heme breathing in cytochromes type *c*), 1126 (bond vibrations in cytochromes type *b*), and 1583 cm^–1^ (vibrations of heme methine bridges in cytochromes). Peaks from proteins (1004 cm^–1^, phenylalanine, and 1654 cm^–1^, amide I) and lipids (1448 cm^–1^, CH_2_ vibrations) in the cytoplasm do not change significantly (Figure 9b). The ratio of peak intensities of reduced cytochromes *c* and *b* to the protein demonstrated the statistically significant drop in the reduced cytochromes in treated cells (Figure 9c,d). That may indicate the redox imbalance in treated cells with prevalence of oxidative processes and may be linked to membrane potential loss.^46,47^ The absence of changes in the ratio of cytochromes *c* to cytochromes *b* indicates the preservation of the stoichiometry of the mitochondrial electron transfer chain complexes (Figure 9e). Cells treated with GalCer fibrils showed no statistically significant differences from the control group in the ratios of cytochromes. Bright-field images of sphingolipid fibrils (Figure 9a­(ii,iii)) treated cells indicate altered cellular morphology as compared to untreated control (Figure 9a­(i)). We aimed to examine whether endogenously formed sphingolipid fibrils are also cytotoxic. For this purpose, we treated the SHSY-5Y cells with 5 μM GALC enzyme inhibitor azo-galacto-fagomine (AGF) (54 ref) for up to 10 days to induce fibril formation. Cell viability was determined on days 5, 7, and 10 using the MTT assay. We observed significant reduction of cell viability with time (Figure S11).

**Figure 9:**
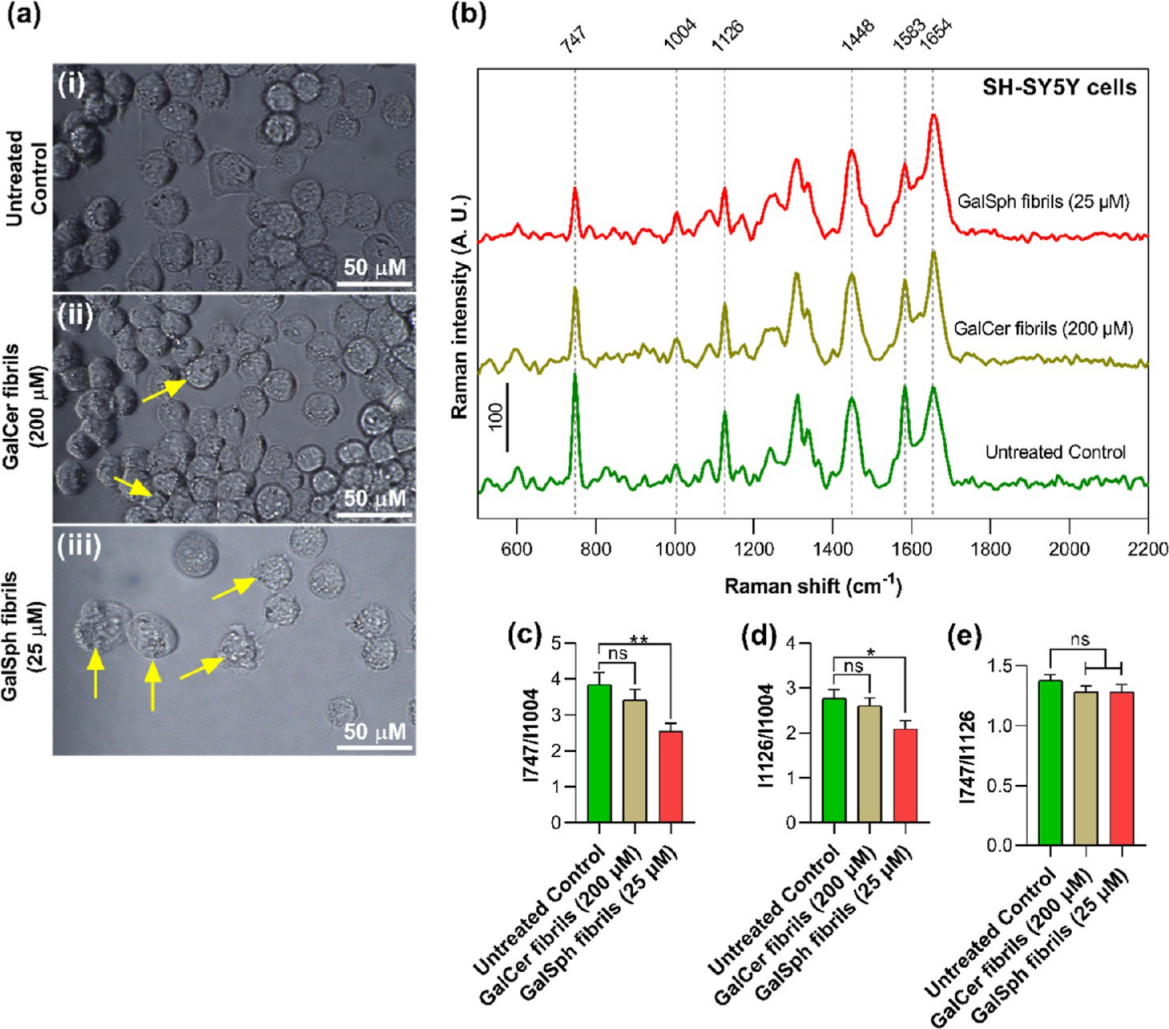
Effect of sphingolipid fibrils on mitochondrial integrity. (a) Bright-field images of control (i) and GalCer-(ii) and GalSph-(iii) fibrils-treated cells. Images were taken at ×60 magnification, to view cellular morphology; the scale bar corresponds to 50 μM. Morphological changes were observed in SH-SY5Y cells treated with GalCer and GalSph fibrils, as indicated by the yellow arrow. (b) Raman spectra of the cytoplasm of SH-SY5Y cells: control (green line), GalCer fibrils (200 μm) (brown line), and GalSph fibrils (25 μm) (red line) after baseline correction. The numbers above peaks represent peak positions. Scale is 100 arbitrary units. (c–e) Raman ratios of peak intensities of control cells and treated cells. (c) Ratio of peak intensities at 747 cm^–1^ corresponding to reduced cytochrome *c* (Fe^2+^) to the peak at 1004 cm^–1^ corresponding to phenylalanine in proteins; (d) ratio of peak intensities at 1126 cm^–1^ corresponding to reduced cytochrome *b* (Fe^2+^) to the protein peak; (e) ratios of peaks from cytochromes *c* and *b*. **p < 0.01, *p < 0.05, ns—not significant by the ANOVA test.

Taken together, these results indicate that cytotoxicity of the sphingolipid fibrils toward the SH-SY5Y cells involved apoptosis and disruption of mitochondrial integrity.

### Mitigation of GalCer and GalSph Fibril Formation In Vitro by Bona Fide Small Molecule Inhibitors

The progressive accumulation of GalCer and GalSph in the lysosome may play a crucial role in GLD pathophysiology, and we observed that upon accumulation, GalCer and GalSph form amyloid-like fibrils in vitro. Previous studies have demonstrated that self-assembly of metabolite amyloid-like fibrils can be inhibited by small molecules that are bona fide inhibitors of proteinaceous amyloids such as NQTrp, Cl-NQTrp, and epigallocatechin-3-gallate (EGCG).^20^ Importantly, efficient mitigation of fibrils of peptides, proteins, and metabolites was not accompanied by significant toxicity of the small molecules to cells at effective concentrations.^48–52^ We tested the effect of these candidate inhibitors toward aggregation of GalCer and GalSph by incubating them with monomeric GalCer or GalSph at various molar ratios (sphingolipid:small molecule 10:1, 5:1, 1:1) in AB (pH 4.5) at 37 °C for 24 h and recorded the end point ThS fluorescence. EGCG, NQTrp, and Cl-NQTrp were found to inhibit the formation of GalCer (Figure 10a­(i–iii)) and GalSph (Figure 11a­(i–iii)) fibrils as reflected by the significant reduction in the ThS fluorescence intensity in comparison to the control samples of monomers without the small molecules. Among the molecules examined, NQTrp was found to cause the greatest reduction of ThS fluorescence indicating the strongest inhibition of fibril formation.

**Figure 10:**
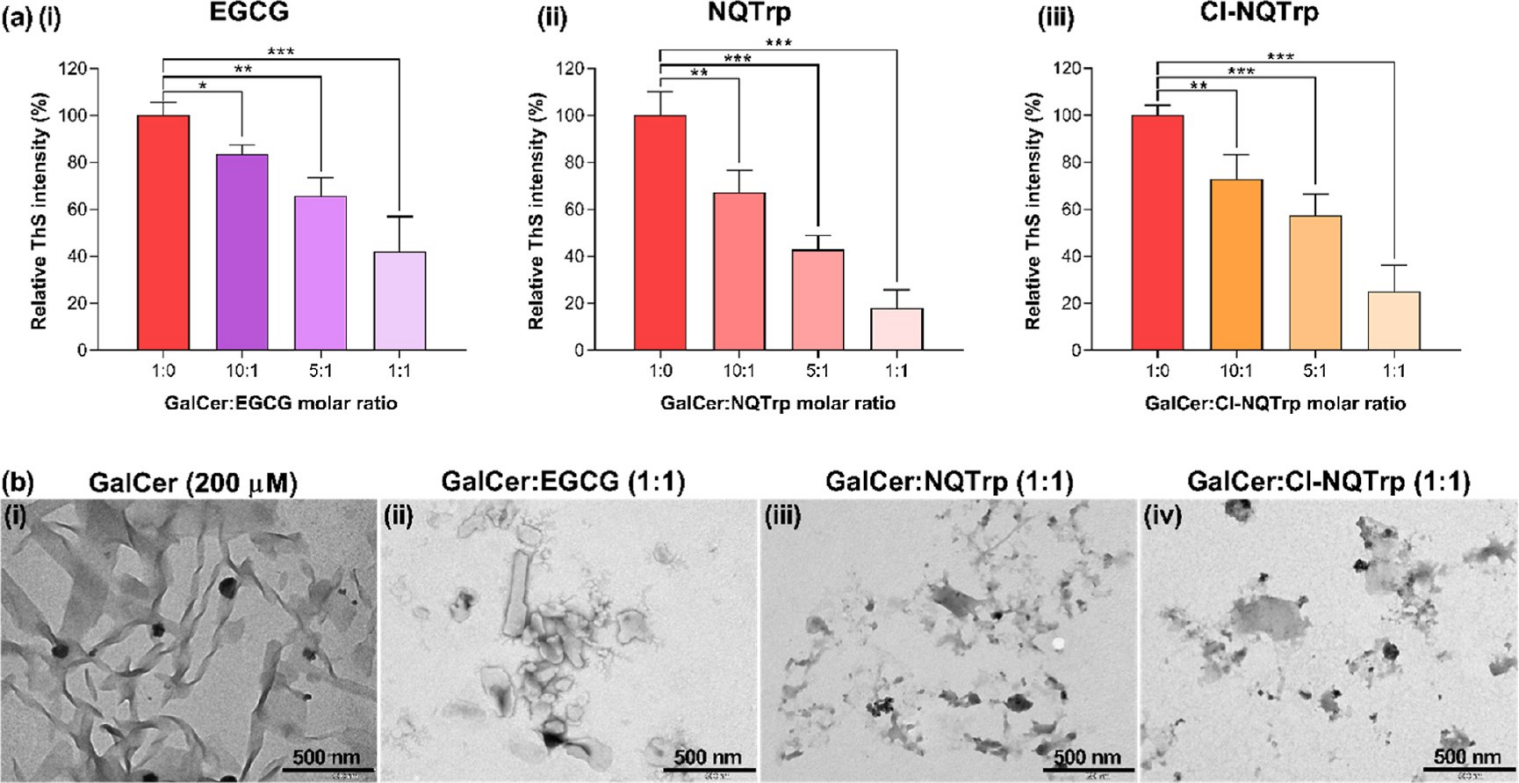
Mitigation of GalCer aggregation by small molecule inhibitors. (a) The normalized ThS fluorescence intensities of GalCer incubated with (i) EGCG, (ii) NQTrp, and (iii) Cl-NQTrp at different molar ratios (10:1, 5:1, and 1:1) show concentration-dependent inhibition of the amyloid-like fibril formation by the inhibitors. The statistical analysis was performed by a Student’s *t*-test to compare untreated control with the inhibitors-treated samples. The asterisks denote statistically significant results (*p < 0.05, **p < 0.01, ***p < 0.001). The data is represented as mean ± SEM (*n* = 3). (b) TEM images of GalCer aggregates in the absence (i) or presence of a 1-fold molar ratio of the inhibitors (ii) EGCG, (iii) NQTrp, and (iv) Cl-NQTrp.

**Figure 11:**
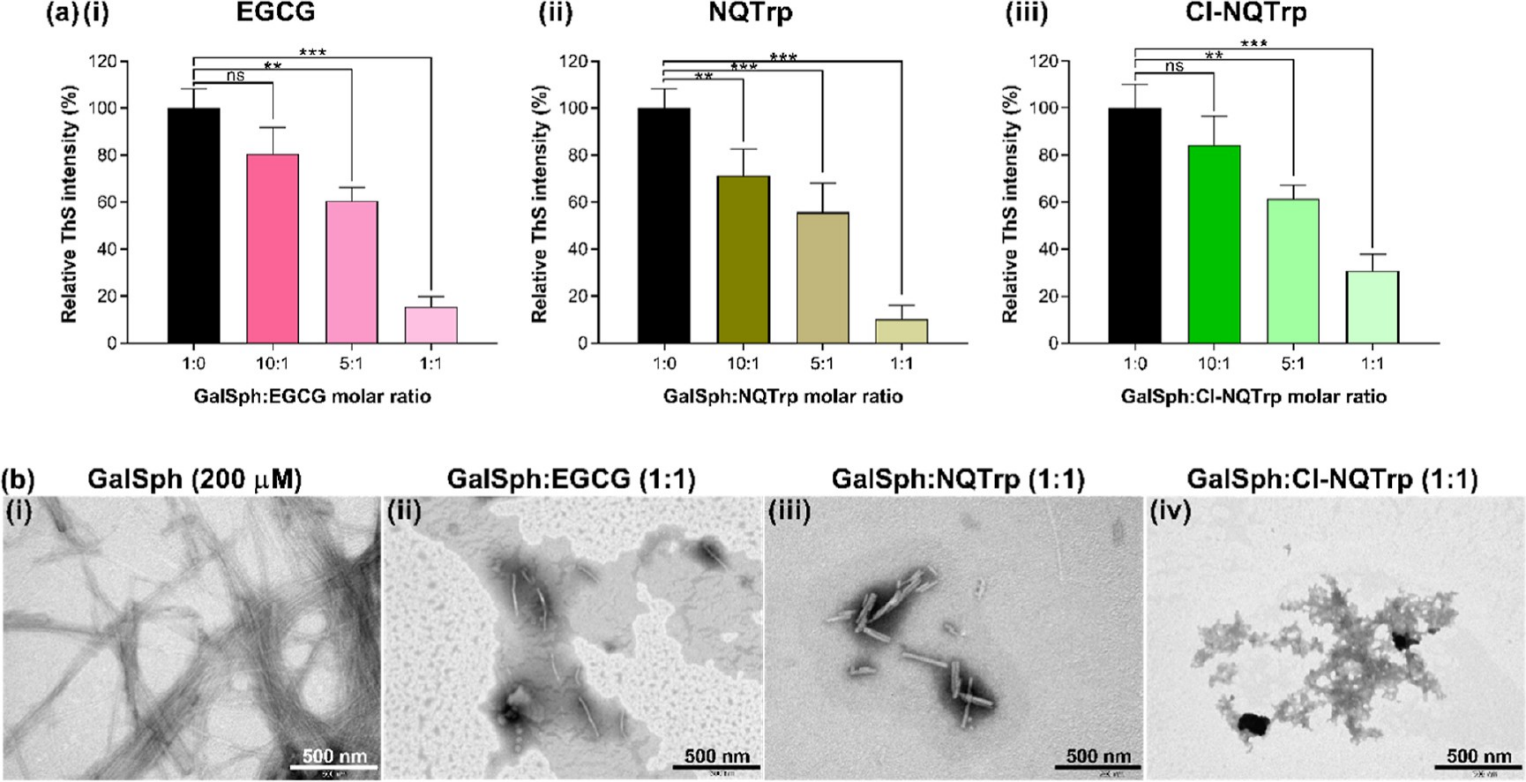
Mitigation of GalSph aggregation by small molecule inhibitors. (a) The normalized ThS fluorescence intensities of GalSph incubated with (i) EGCG, (ii) NQTrp, and (iii) Cl-NQTrp at different molar ratios (10:1, 5:1, and 1:1) show concentration-dependent inhibition of the amyloid-like fibril formation by the inhibitors. The statistical analysis was performed by a Student’s *t*-test to compare untreated control with the inhibitors-treated samples. The asterisks denote statistically significant results (*p < 0.05, **p < 0.01, ***p < 0.001). The data is represented as mean ± SEM (*n* = 3). (b) TEM images of GalSph aggregates in the absence (i) or presence of a 1-fold molar ratio of the inhibitors (ii) EGCG, (iii) NQTrp, and (iv) Cl-NQTrp.

We corroborated these findings by TEM analysis of the samples after 24 h of incubation. No fibrillary structures were formed in the mixture of GalCer and GalSph with either EGCG, NQTrp, or Cl-NQTrp, whereas the control samples of GalCer (Figure 10b­(i–iv)) and GalSph (Figure 11b­(i–iv)) without an inhibitor exhibited ample fibrils. Based upon these findings, we chose NQTrp for further experiments.

### NQTrp Reduces Sphingolipid Fibril Formation and Mitigates Fibril-Induced Cytotoxicity in Neuronal Cells

Given that amyloid inhibitors such as NQTrp exhibited an inhibitory effect toward GalCer (Figure 10a­(ii),b­(iii)) and GalSph (Figure 11a­(ii),b­(iii)) fibril formation in vitro and ameliorated their toxicity to cells in culture, we next examined whether NQTrp can inhibit fibril formation of these sphingolipids within the cell. For this purpose, we induced accumulation and fibril formation of GalCer and GalSph in SH-SY5Y cells by using a GALC enzyme inhibitor AGF.^53^ First, we evaluate the desired dose of AGF for inducing endogenous sphingolipid accumulation; we incubated the cells with different doses of AGF (0.5–50 μM) for five consecutive days after which we monitored the abundance of GalCer puncta by immunofluorescence staining using anti-GalCer antibodies. AGF concentration of 5 μM or above resulted in overt GalCer puncta inside the cells (Figure S12). Therefore, we chose 5 μM AGF for further experiments. The SH-SY5Y cells were incubated with 5 μM AGF for 5 days, and on day 6, different molar concentrations of NQTrp (5–100 μM) were added to the culture medium and incubated for an additional 48 h, after which the presence of GalCer puncta was examined. Cells treated with neither AGF nor NQTrp and cells treated with AGF only served as controls. Cells incubated with AGF contained only ample GalCer-positive puncta, suggesting abundant fibrils (Figure 12a–h). A dose-dependent significant reduction in the abundance of GalCer puncta with increasing NQTrp concentration was observed, as quantified by ImageJ (Figure 12i). These observations indicate that a bona fide inhibitor of aggregation of amyloidogenic proteins, namely, NQTrp, can mitigate the formation of fibrils of GalCer in cells.

**Figure 12:**
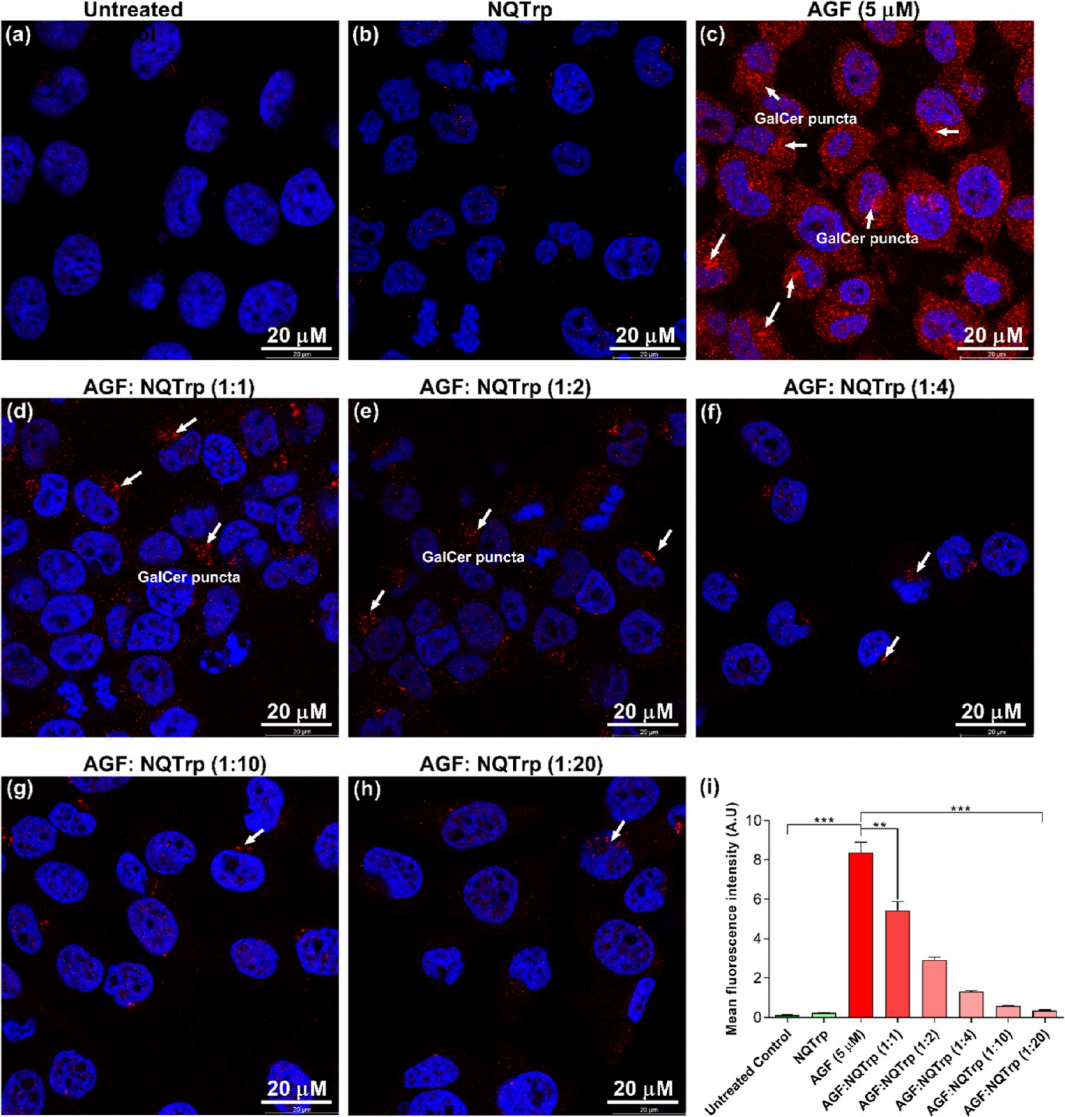
Effect of NQTrp on mitigation of endogenous GalCer accumulation in SH-SY5Y cells treated with the GALC inhibitor. Cells were treated with AGF for 5 days and then incubated with different molar ratios of NQTrp. GalCer accumulation in SH-SY5Y cells was detected using a GalCer-specific antibody. Red puncta inside the cells indicate accumulation of GalCer in (a) untreated control, (b) NQTrp-treated, and (c–h) GALC inhibitor-treated cells in the absence (c) or presence (d–h) of NQTrp at various molar ratios. (i) ImageJ quantification of GalCer puncta in SH-SY5Y cells. Values are represented as mean ± SEM (*n* = 6). The data were analyzed using one-way ANOVA followed by the Tukey multiple comparison posthoc test. The asterisks denote statistically significant results (**p < 0.01, ***p < 0.001).

We next investigated the potential of NQTrp to inhibit the cytotoxicity induced by GalCer and GalSph fibrils toward SH-SY5Y cells. For this analysis, NQTrp was mixed into monomeric solutions of GalCer and GalSph, which were prepared in the culture medium. SH-SY5Y cells were exposed to the GalCer and GalSph monomers for 24 h, in either the presence or absence of the inhibitor. MTT assay results demonstrated a significant increase in cell viability upon treatment with NQTrp (Figure 13a,b). We next examined the effects of NQTrp on GalSph fibrils-induced cellular apoptosis and mitochondrial redox homeostasis in SH-SY5Y cells. This experiment followed the same treatment protocol as mentioned in the MTT assay and was performed with GalSph fibrils since they exhibited stronger cytotoxicity than GalCer fibrils (Figure 6). The results indicated that the GalSph fibril-treated cells displayed a significant increase in apoptotic cell death (∼28 ± 2.01%) as compared to untreated cells (∼9 ± 1.4% apoptotic cells) (Figure 14a–h). Incubation of the GalSph fibrils with NQTrp (molar ratios 1:1 and 1:2) significantly reduced the apoptotic (∼20 ± 1.51% and ∼17 ± 1.7%, respectively) cell death as compared to GalSph fibril treated cells without the inhibitor (Figure 14f–h). We did not observe any significant changes in the 5:1 and 2:1 molar ratio of GalSph fibrils:NQTrp (Figure 14d–h). Cells exposed to 25 μM GalSph fibrils displayed a marked decrease in the MMP, indicating a transition of cell population JC-1 red (aggregates of JC-1, ∼32 ± 1.54%) to JC-1 green (monomers of JC-1, ∼67 ± 1.59%) fluorescence. NQTrp (1:1 and 1:2 molar ratios) significantly improved GalSph fibril-induced impairments in the MMP (Figure 15a–g). The quantification of the percentage of JC-1 monomer cell population significantly reduced upon 1-fold (∼43 ± 1.2%) and 2-fold (∼23 ± 1.19%) NQTrp treatment (Figure 15h). These results suggested that NQTrp attenuates GalSph fibril-induced mitochondrial dysfunction, and such effects could play an important role in protecting SHSY5Y cells against cytotoxicity of these fibrils.

**Figure 13:**
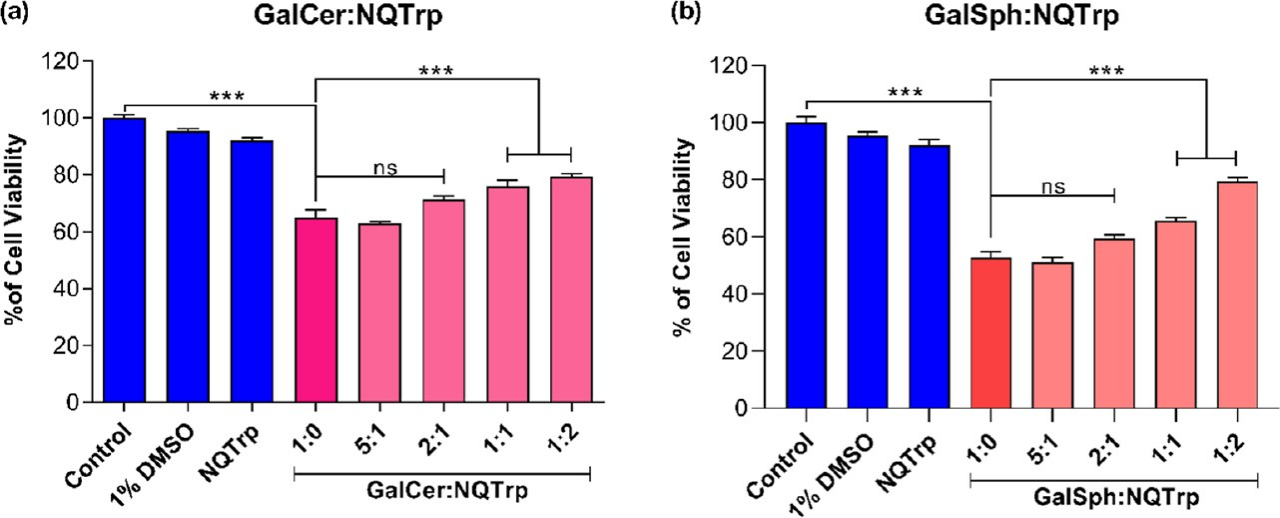
Effect of NQTrp on GalCer and GalSph fibrils-induced cytotoxicity in SH-SY5Y cells. Cells were exposed to (a) GalCer and (b) GalSph fibrils without or with different molar ratios (5:1, 2:1, 1:1, 1:2, and 1:5) of NQTrp for 24 h, and cell viability was measured by MTT assay. Values are represented as mean ± SEM (*n* = 6). Significance was analyzed using one-way ANOVA followed by the Tukey multiple comparison test, *p < 0.05, **p < 0.01, ***p < 0.001.

**Figure 14:**
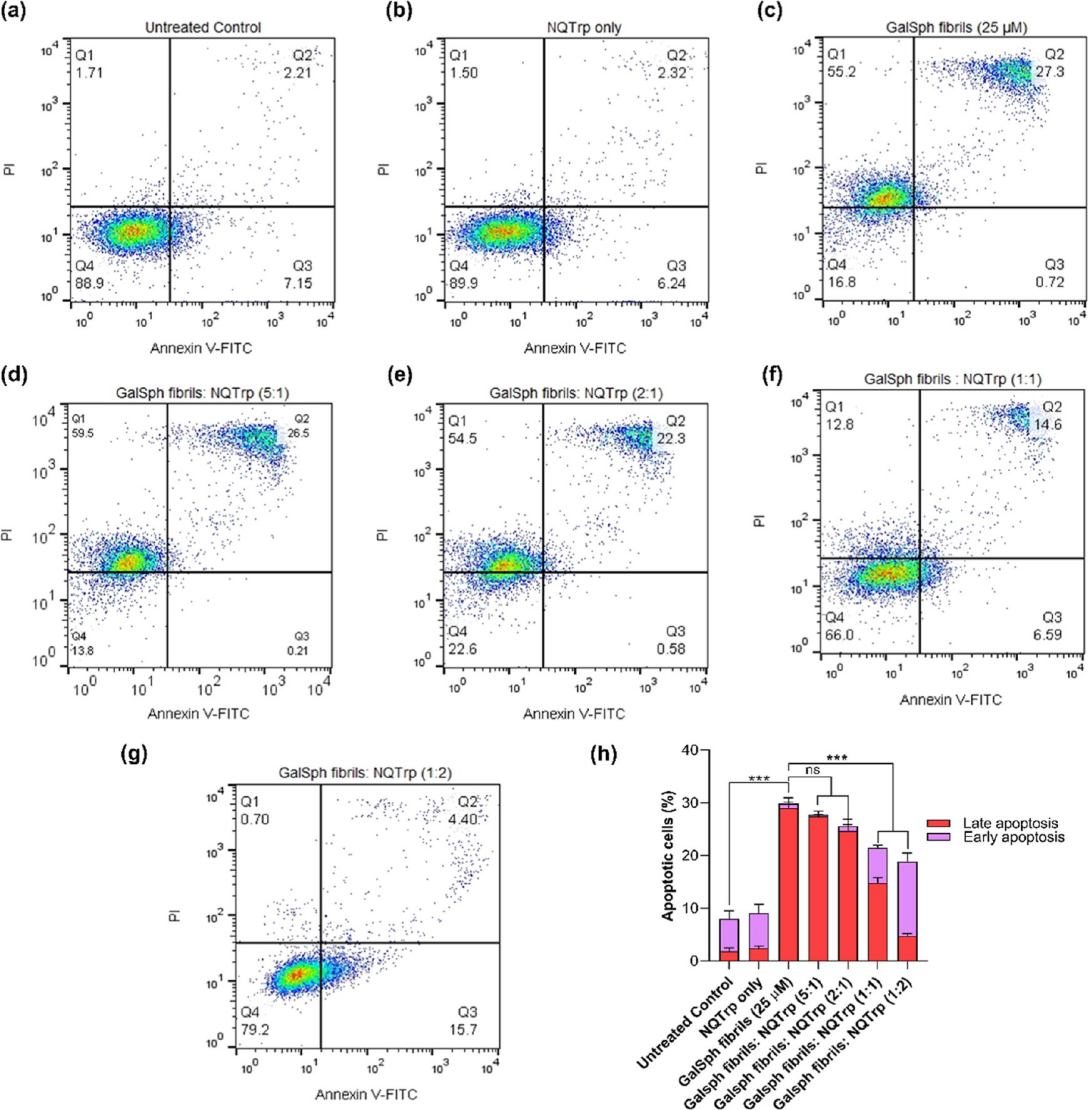
Effect of NQTrp on GalSph fibrils-induced cellular apoptosis in SH-SY5Y cells. Flow cytometry analysis of cell death in annexin V- and PI-stained cells showing the percentage of necrotic, late apoptotic, early apoptotic, and viable cell populations in SH-SY5Y cells. Cells were exposed to GalSph fibrils without (c) or with different molar ratios (5:1, 2:1, 1:1, and 1:2; (d), (e), (f), and (g), respectively) of NQTrp for 24 h. Untreated (a) and NQTrp only (b)-treated cells serve as controls. (h) Bar plot showing quantification of GalSph fibrils-induced apoptotic events in cells treated with or without NQTrp. Values are represented as mean ± SEM, *n* = 6. Significance was analyzed using one-way ANOVA followed by the Tukey multiple comparison test, *p < 0.05, **p < 0.01, ***p < 0.001.

**Figure 15:**
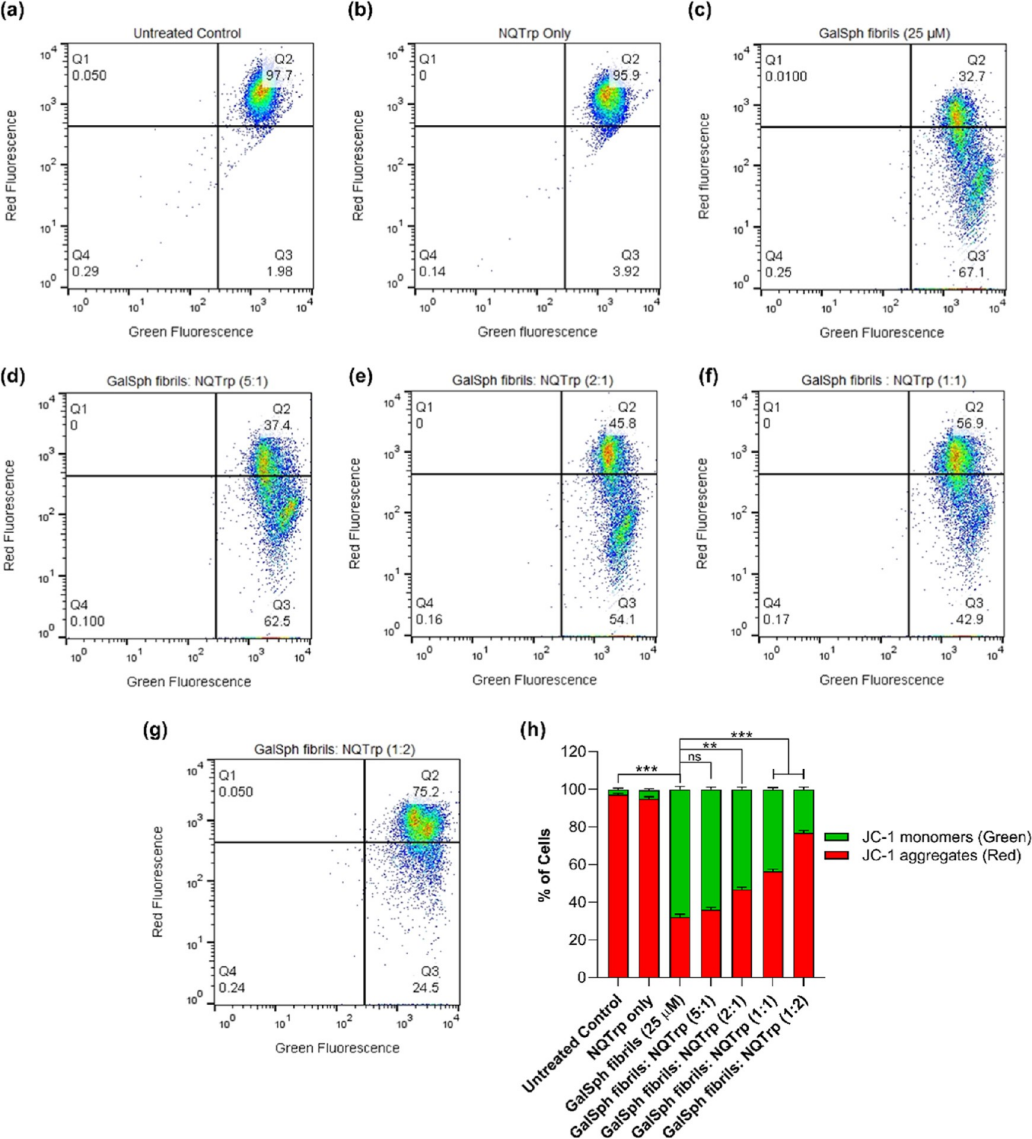
Effect of NQTrp on GalSph fibrils-induced loss of MMP in SH-SY5Y cells. Cells were exposed to GalSph fibrils without (c) or with different molar ratios (5:1, 2:1, 1:1, and 1:2; (d), (e), (f), and (g), respectively) of NQTrp for 24 h. Untreated (a) and NQTrp only (b)-treated cells serve as controls. The scatter plot represents JC-1 red fluorescence (aggregates) versus JC-1 green fluorescence (monomers), indicating mitochondrial membrane potential. A shift toward increased green fluorescence signifies mitochondrial depolarization. The plot shows JC-1 red versus JC-1 green emission from flow cytometry on mitochondrial membrane potential. Increased green fluorescence (cells shift toward the green channel) indicates depolarized mitochondria. (h) The bar graph represents the quantification of JC-1 monomer-to-aggregate in GalCer- and GalSph-treated cells with or without NQTrp incubation, expressed as the percentage of cells in each condition. Values are mean ± SEM, *n* = 3. Statistical significance was analyzed using one-way ANOVA followed by the Tukey multiple comparison posthoc test, **p < 0.01, ***p < 0.001 vs untreated control.

## Discussion

The amyloidogenic self-assemblies of proteins and peptides have been linked with several human diseases such as Creutzfeldt–Jakob disease, Parkinson’s disease, Alzheimer’s disease, amyotrophic lateral sclerosis, and type 2 diabetes.^54,55^ However, a new paradigm has emerged in light of recent discoveries, suggesting that the self-assembly into amyloid-like fibrils is not limited to proteins but can also involve certain metabolites.^56^ The propensity of metabolites to undergo ordered amyloid-like aggregation in vitro constitutes a substantial augmentation of the “amyloid hypothesis” and introduces a new paradigmatic perspective on the etiology of inborn errors of metabolism disorders. The current study offers both in vitro and in vivo demonstration and key experimental tools for the study of metabolite sphingolipids aggregation phenomena associated with GLD.^5^

In GLD, two major sphingolipid substrates GalCer and GalSph, components of myelin lipids, accumulate in macrophage cells which further infiltrate into the brain parenchyma resulting in damage of myelin sheath, dysfunction of oligodendrocytes, and disruption of axonal transport.^10,57,58^ Sphingolipid accumulation is linked to two primary metabolic pathways. The first is a synthetic pathway, where ceramide galactosyltransferase catalyzes the addition of a β-d-galactosyl group to sphingosine.^59,60^ The second pathway involves the degradation of galactosylceramide (GalCer) by acid ceramidase, which hydrolyzes ceramides into sphingosine and fatty acids.^61^ It is also reported that GALC deficiency results in the accumulation of GalSph as a primary metabolite and, to a lesser extent, GalCer.^62^ The precise mechanisms underlying sphingolipid-induced toxicity in GLD remain poorly understood. Detailed structural analysis is essential to elucidating the interactions and effects of GalSph and GalCer on cellular components and myelin integrity.

In the present work, our results demonstrate that monomeric GalCer and GalSph can self-assemble spontaneously in vitro into highly ordered structures reminiscent of proteinaceous amyloids. Complementary in vitro biophysical techniques including ThS kinetics, CR-birefringence, and ANS assay confirmed the amyloidogenic properties of the GalCer and GalSph self-assemblies. Binding of these amyloid-specific dyes with these sphingolipid aggregates indicates the existence of hydrophobic structures as present in the proteinaceous amyloid. The morphology of these structures resembled typical amyloid-like fibrils. Our work further demonstrated that both GalCer and GalSph can form amyloid-like fibrils in vitro under physiologically relevant conditions (pH 7.4) as well as under acidic conditions (pH 4.5) mimicking the lysosomal milieu where these metabolites accumulate in GLD. These assemblies are not amorphous aggregates; rather, they have fibrillar morphology that differs between these two sphingolipids. TEM images indicated that GalCer fibrils are flat or twisted helical ribbons with a diameter of ∼40 nm, comparable to those reported for the GlcCer metabolite which accumulated in GD, whereas GalSph fibrils are straight long fibrils with a diameter of ∼9.2 nm. We speculate that the double-chain ceramide moiety may have facilitated the formation of the twisted fibrillary structure in the GalCer and GlcCer assemblies, whereas the single-chain sphingosine of GalSph may render the formation of the straight-chain filament-like structure. These structural features are similar to the previously reported structures reported from GLD patient brain samples.^63^ In these aspects, the properties of these fibrils resemble those of proteinaceous amyloid assemblies, which are related to numerous pathological disorders. It remains to be examined whether other sphingolipids can self-assemble into amyloid-like fibrils. Many inherited metabolic diseases arise from mutations that disrupt a metabolic enzyme, resulting in its misfolding, functional impairment, and subsequent accumulation of metabolites. Accumulated metabolites formed higher-order structures comparable to amyloid fibrils, and could induce cytotoxicity as known for other amyloid assemblies.^64–67^

FTIR and Raman microspectroscopy were used to characterize the vibrational spectroscopic properties of GalCer and GalSph monomers and fibrils, providing both chemical and structural information. In FTIR studies, the fibrils of the lipids showed a noticeable downshift of amide I and amide II peak values in comparison to their monomers, clearly suggesting the fluid-like monomeric lipids transform into the arranged H-bonded lamellae phase, which are gel-like fibrillary structures.^20,32^ In addition, we observed another visible downshift in the FTIR peaks corresponding to the symmetric and asymmetric stretching vibration of the ceramide/sphingosine CH_2_ groups, suggesting the conversion of the fluid-like monomeric to gel or crystalline phase of the fibrillar lipids. These FTIR results corroborated Raman microspectroscopic results. These spectroscopic changes observed during fibril formation are due to additional intermolecular interactions (H-bonding and nonpolar interactions) compared to the monomeric compounds. The greater the number of intermolecular interactions and the higher the level of structural organization in fibrils, the more peaks that get resolved and they are typically sharper, especially in the case of GalSph. A schematic diagram for the formation of sphingolipid fibrils is shown in Figure 16. In Raman spectra, certain covalent chemical bonds preferentially vibrate in a parallel orientation relative to the laser polarization, resulting in an enhanced intensity for those bonds. This polarization characteristic is typically seen in highly oriented crystalline materials, providing further evidence of the formation of fibrillar aggregates. To summarize the three main observations when comparing monomeric and fibrillar forms, we can distinguish them based on multiple parameters: (i) Monomeric material does not polarize the scattered signal due to the lack of stable structural organization. (ii) Spectral differences, such as the presence or absence of peaks, changes in intensity proportions, and spectral shifts. (iii) Sharper peaks, indicating higher crystallinity, compared to the broader, unresolved peaks of less organized or amorphous material (Figure 4e). This effect is particularly pronounced in the case of GalSph and will require further investigation in future studies. Our cumulative results confirm GalCer and GalSph fibrillar structures as toxicity-causing agents. Time-dependent MTT assay data indicated monomeric GalCer and GalSph were nontoxic at the initial period (up to 6 h) but after prolonged incubation (up to 12 h) became toxic toward SH-SY5Y cells. Our results also demonstrated that GalSph fibrils are more toxic than GalCer fibrils. The possible explanation could be due to the different morphologies of these fibrils. GLD has previously been shown to be associated with the elevation of GalSph concentrations and its toxicity.^68^ The toxicity of GalSph has been a focus for various studies yet is still incompletely understood. One proposed mechanism is the disruption of cellular and myelin membranes.

**Figure 16:**
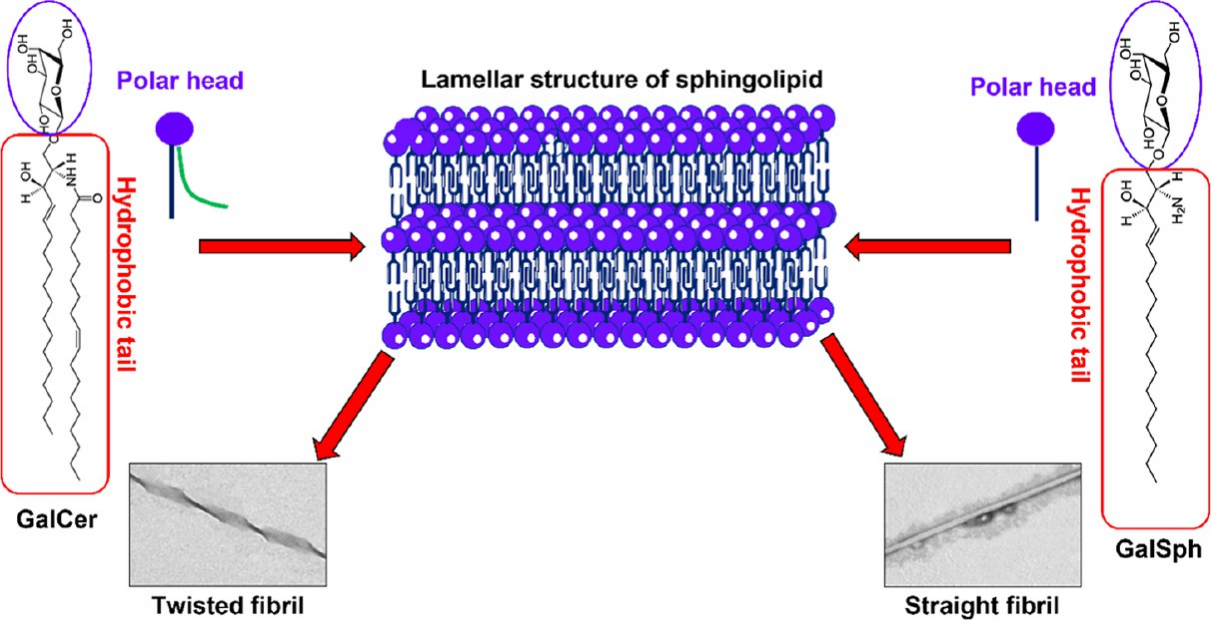
A schematic diagram representing a possible mechanism of sphingolipid fibril formation.

The present work provides an additional perspective, based on our finding of supramolecular structures of GalSph, and GalCer self-assemblies, which are cytotoxic and become abundant when GALC activity is disrupted, as in GLD. An initial insight into the underlying mechanism of toxicity was obtained by our observation that the fibrils of both GalCer and GalSph disrupt mitochondrial function, as indicated by a loss of mitochondrial membrane potential. This depolarization of the mitochondrial membrane triggers the activation of apoptotic pathways. Use of the direct nondestructive Raman microspectroscopy allowed quantification of the effect of the fibrils on the mitochondrial redox state, as evidenced by an oxidation of cytochrome *c*, which is indicative of mitochondrial dysfunction and apoptosis initiation, supporting the hypothesis that GalSph-induced mitochondrial dysfunction plays a central role in driving cell death in GLD.

Mitigation of amyloid formation is an attractive therapeutic strategy for various amyloid-associated diseases.^20,69^ Here, by using small antiamyloid inhibitors (EGCG, NQTrp, and Cl-NQTrp), we found efficient inhibition of GalCer and GalSph aggregation in vitro. The underlying inhibitory mechanism toward proteinaceous amyloids by such small molecules, which commonly comprise aromatic moieties, has been facilitated by hydrogen bond formation and π–π stacking with the aromatic amino acids of the target amyloid, resulting in the breakdown of fibrillar architecture.^70^ The mechanism of the effect of these small molecules on nonproteinaceous fibrils is presently unknown. In this investigation, we demonstrated that GalCer and GalSph fibrils induce cytotoxicity in SH-SY5Y and oligodendroglial cells in a dose-dependent manner. Our data show a decrease in mitochondrial membrane potential and oxidation of cytochromes in the electron transport chain, suggesting mitochondrial dysfunction. While we did not measure ROS directly, these findings are consistent with a mechanism involving oxidative stress and disruption of mitochondrial integrity, which may ultimately lead to cytochrome *c* release, and activation of the apoptotic pathway. GLD treatment presents a significant medical challenge, as current treatment options, including gene and cell therapy, face hurdles in achieving sustained GALC levels, particularly within critical organs such as the central and peripheral nervous system. Our results point to the self-assemblies of GalCer and GalSph as possible novel targets for GLD therapeutics and provide initial evidence of their mitigation using small molecules.

## Conclusion

In the present study, our results showed that GalCer and GalSph exhibit self-assembly into fibrillar structures in vitro, which are highly reminiscent of proteinaceous amyloids as well as of fibrils of GlcCer, which accumulates in Gaucher disease. The kinetics of sphingolipid aggregation, monitored using several amyloid-specific dyes, including ThS, ANS, and CR binding assays, indicate the presence of hydrophobic interaction and ordered structures. Morphological analysis using TEM revealed that GalCer forms twisted and flat ribbon-like fibrils, whereas GalSph forms thinner, straighter fibrils resembling amyloid-β or α-synuclein aggregates. Furthermore, these fibrils remain stable over extended incubation periods and across various temperatures, suggesting a robust and persistent supramolecular structure.

Spectroscopic analyses, including FTIR and Raman microspectroscopy, indicate extensive intermolecular hydrogen bonding and ordered packing. PXRD and SAXS further confirm the presence of a gel-phase, bilayer arrangement, similar to the reported GlcCer fibrils. PXRD and SAXS analyses indicate a typical repeating correlation length present in GalCer compared to GalSph fibrils. This may be due to the different functional groups and fatty acid chain lengths of the two sphingolipids. These findings provide critical insights into the molecular architecture of sphingolipid aggregates and their potential implications for GLD pathology.

The cytotoxicity of GalCer and GalSph fibrils toward neuronal (SH-SY5Y) and oligodendrocyte (DDR1) cells was evident as a dose-dependent decline in cell viability, with GalSph fibrils exhibiting markedly higher toxicity than GalCer. Flow cytometric analysis confirmed the induction of apoptosis, as evidenced by the increased annexin V- and PI-positive cell populations. JC-1 staining further revealed a disruption of the mitochondrial membrane potential, indicative of mitochondrial dysfunction. Raman microspectroscopic analysis identified a reduction in cytochrome signals, suggesting oxidative stress and redox imbalance, particularly in GalSph-treated cells. Notably, amyloid inhibitors such as NQTrp, EGCG, and Cl-NQTrp effectively suppressed fibril formation in vitro, with NQTrp demonstrating the most potent inhibition. Furthermore, NQTrp significantly mitigated fibril-induced cytotoxicity and apoptosis and restored mitochondrial function, highlighting its potential as a therapeutic intervention. These findings suggest that GalCer and GalSph fibrils contribute to GLD pathology via apoptotic cell death and mitochondrial impairment and that their cytotoxic effects may be attenuated through targeted amyloid inhibition strategies.

## Experimental Section

### Materials

All reagents and chemicals used for the study were purchased from Sigma-Aldrich (Rehovot, Israel) unless mentioned otherwise. β-Galactosylceramide (GalCer, Cat. No: 860596P) and galactosylsphingosine (GalSph, Cat. No: 860537P) were purchased from Avanti Polar lipids (AL, USA). Molecular biology grade ultrapure water Dulbecco phosphate buffer saline (DPBS, 1×) and cell culture reagents were purchased from Sartorius (Beit HaEmek, Israel).

### Stock Sample Preparation

GalCer and GalSph powders were each dissolved in 100% dimethyl sulfoxide (DMSO) to prepare 10 mM stocks, which were further diluted to desired concentrations with either phosphate buffer saline (PBS) of pH 7.4, acetate buffer (AB) of pH 4.0, or double distilled water (DDW), and were used as required. The working concentration of the buffer system for all of the assays was 100 mM. A freshly prepared stock solution of the sphingolipids was used before each assay. Stock solution of ThS (4 mM) was prepared in DDW. Stock solutions of the tested inhibitor molecules (10 mM) were each prepared in DMSO and diluted with either PBS or AB before use.

### Thioflavin S Fluorescence Assay

ThS fluorescence assay was carried out to monitor the aggregation kinetics of GalCer and GalSph in a similar manner as described previously for monitoring aggregation of GlcCer^20^ and amyloidogenic proteins.^51^ Briefly, the stock solutions of the two sphingolipids were diluted in 200 μL wells of a 96-well black plate (Corning, NY, USA) so that the final mixture contained 20–300 μM of GalCer and GalSph and 30 μM ThS in 100 mM of either PBS, AB, or DDW. The samples were allowed to aggregate for 24 h at 37 °C with shaking for 30 s before each measurement. ThS fluorescence intensity was recorded using an Infinite M200 microplate reader (Tecan, Mannedorf, Switzerland) at 485 nm after exciting the dye at 440 nm, with measurements taken at 5 min intervals. For each time point, three recorded values were averaged, and background measurements of buffer containing only ThS fluorescence intensity were subtracted.

For monitoring the inhibition of GalCer and GalSph aggregation, small molecule inhibitors were incubated with monomeric GalCer and GalSph at different molar ratios (sphingolipids:inhibitor = 10:1, 5:1, and 1:1) for 24 h at 37 °C on a rotating platform. The GalCer and GalSph, without any inhibitors, as well as each inhibitor without the sphingolipids, were also incubated and served as control groups. Subsequently, the samples were transferred to a black 96-well plate and mixed with 30 μM ThS. The fluorescence intensity was recorded using the above-mentioned experimental conditions, and the reduction in ThS fluorescence intensity was monitored.

### Turbidity Assay

Stock solutions of 10 mM GalCer or GalSph were prepared by dissolving the corresponding sphingolipid powder in 100% DMSO. The stock solutions were diluted to various concentrations with PBS (or AB) in polypropylene 96-well plates (Corning, NY, USA) to a final volume of 200 μL and incubated at 37 °C. The plates were shaken gently for 5 s prior to each measurement. The turbidity of the solution was monitored by recording the optical density (OD) at 350 nm using an Infinite M200 microplate reader (Tecan, Mannedorf, Switzerland) at time zero (*T*_0_) and after 24 h of incubation. OD of the buffer at 350 nm was subtracted in order to eliminate background absorbance.

### 8-Anilinonaphthalene-1-sulfonic acid Fluorescence Assay

A 20 μL aliquot of GalCer or GalSph aggregates taken from the end point of the turbidity assay was mixed with 80 μM of 8-anilinonaphthalene-1-sulfonic acid (ANS) from a 5 mM ANS stock in the desired buffer (PBS or AB). ANS fluorescence intensity was measured with excitation at 380 nm and emission between 400 and 700 nm using an Infinite M200 microplate fluorescence reader (Tecan, Mannedorf, Switzerland).

### Transmission Electron Microscopy

The morphology of the GalCer and GalSph aggregates was assessed by TEM. Samples (5 μL) from the end of the turbidity assay were drop-cast on a 400-mesh carbon stabilized Formvar coated copper grid (Ted Pella, Inc., California, USA). Excess fluid was removed using a lint-free paper. Thereafter, the sample was negatively stained using 5 μL of 2% (w/v) phosphotungstic acid (PTA) for 40 s, and the extra stain was removed as above. The grids were then dried overnight at room temperature (RT) and the morphology of the aggregates was visualized using a JEM-1400 electron microscope (JEOL, Tokyo, Japan) accelerating at 80 kV.

### Congo Red Birefringence

The GalCer and GalSph aggregates were generated by incubating the respective monomers (from 10 mM DMSO stock) in PBS (pH 7.4) at a concentration of 200 μM and then were mixed with Congo red (CR) solution (5 mM stock in DDW) in a flat-bottomed, transparent 96-well plate (Corning, New York, USA). The final concentration of CR was set at 50 μM. Subsequently, the mixture was incubated for 15 min at RT. The suspension (10 μL) was then drop cast on a glass side, and the samples were air-dried and kept in a desiccator before birefringence analysis. The samples were viewed at 20× magnification with a Nikon Eclipse TI polarizing microscope. Digitized images were obtained by using a Nikon DS Ri1 digital camera. CR alone served as the negative control.

### ThS Staining and Fluorescence Imaging

Samples of GalCer and GalSph fibrils (10 μL), taken from the end point of the turbidity assay, were mixed with 10 μL of ThS dye (30 μM). The mixture was drop cast on a glass slide, followed by drying under ambient conditions for 12 h. The dried samples were viewed under a Leica SP8 LIGHTNING (Wetzlar, Germany) confocal system using the built-in software Leica Application Suite X 3.5.5.19976. Images were taken using a 20× dry objective with a numerical aperture of 0.75 and an excitation wavelength of 405 nm. The emission spectra were collected between 422 and 525 nm with 1 pinhole airy. The buffer PBS (pH 7.4) alone mixed with ThS served as the negative control.

### Fourier-Transform Infrared Spectroscopy

Fourier-Transform Infrared (FTIR) spectroscopy was performed with 30 μL of GalCer and GalSph samples (monomers or fibrils) of 200 μM concentration. Monomers were obtained by dissolving GalCer and GalSph powder in a chloroform/methanol (2:1) solvent. Aggregates of GalCer and GalSph were prepared in the same manner as that described above for the turbidity assay. The samples were deposited onto disposable KBr IR sample cards (Sigma-Aldrich, Rehovot, Israel), which were then allowed to dry under a vacuum. Transmission infrared spectra were collected using a Nexus 470 FTIR spectrometer (Nicolet, Thermo Fisher Scientific, MA, USA) with a deuterated triglycine sulfate detector. Measurements were performed using the atmospheric suppression mode by averaging 64 scans in 2 cm^–1^ resolution. The single data have been exported in Excel, normalized, and plotted by Origin 2018.

### Raman Microspectroscopy

Raman spectroscopic characterization was performed on monomeric and fibrillar GalCer and GalSph, prepared as described above. The spectra were recorded using WITec alpha 300 (WITec, Ulm, Germany) equipped with Nikon CFI Plan Fluor 60× NA = 0.85 objective, 532 nm excitation laser with 60 mW power output, UHTS300S detector using 600 gr/mm dispersion grating, 0.5 s accumulation time, and 50–500 accumulations. Data were processed on WITec Project 6.0 software (WITec, Ulm, Germany) using cosmic ray removal, spectra cropping, polynomial and shape background subtraction, and the spectral decomposition tool as described.^71^ The single spectra have been exported, normalized, and plotted by SigmaPlot 12.5.

### Powder X-ray Diffraction

Powder X-ray Diffraction (PXRD) of aggregated GalCer and GalSph was measured as previously described.^20^ Briefly, powdered samples of GalCer and GalSph aggregates were introduced into a 0.5 mm diameter glass capillary for measurement. The aggregates were prepared by incubating 1 mM GalCer and GalSph in deionized distilled water (DDW) for 24 h, followed by lyophilization to obtain a fluffy powder. XRD data were acquired in symmetric Bragg–Brentano geometry with a Cu Kα radiation source (wavelength of 1.54 Å) on a Bruker D8 Discover X-ray diffractometer equipped with a one-dimensional LynxEye detector based on compound silicon strip technology.

### Small-Angle X-ray Scattering

The samples of aggregated GalCer and GalSph, at 5 mM concentration in PBS (pH 7.4), were measured in 1.5 mm diameter sealed quartz capillaries. SAXS measurements were performed using an in-house solution X-ray scattering system, with a Genix 3D (Xenocs) low-divergence Cu Kα radiation source (wavelength of 1.54 Å) and a scatterless slits setup.^72^ Two-dimensional scattering data with a wave vector amplitude (*Q*) range of 0.005–0.4 Å^–1^ at a sample-to-detector distance of about 700 mm were collected on an Eiger2 1 M (Dectris, PA, USA) and radially integrated using MATLAB (MathWorks)-based procedures (SAXSi). The exact sample-to-detector position was calibrated using silver behenate powder. Acquisition time was typically 1800 s per frame. Background scattering data were collected from a buffer solution (PBS, pH 7.4) alone. For each sample, peak position was extracted from the 1D radial profiles.

### Cell Viability Assay

Human neuroblastoma SH-SY5Y cells were cultured in DMEM:nutrient mixture F12 (Ham’s) (1:1) (cat. no. 01-170-1A, Sartorius, Beit HaEmek, Israel) and DDR1 oligodendrocyte cells were maintained in DMEM (cat. no. 01-052-1A, Sartorius, Beit HaEmek, Israel), supplemented with 10% fetal bovine serum (FBS, Cat. No. A5256701, Gibco, MA, USA) and 1% antibiotic (streptomycin and penicillin, cat. No. P4333, Sigma-Aldrich, MO, USA) at 37 °C in a humidified chamber with 5% CO_2_. The cells were trypsinized (cat. no. 03-079-1A, Sartorius, Beit HaEmek, Israel) and seeded in a 96-well plate at 1 × 10^4^ cells per well and incubated overnight. The stock solution of GalCer and GalSph (in DMSO) was dissolved in DMEM:nutrient mixture F12 (Ham’s) (1:1) and DMEM at different concentrations (1–200 μM) for 24 h to allow self-assembly into GalCer and GalSph fibrils. DMSO (1%) was used as the vehicle. Untreated cells served as a negative control. The medium (100 μL) with or without the sphingolipid aggregates was added to each well. Cell viability was determined by the MTT (3-(4,5-dimethyl-2-thiazolyl)-2,5-diphenyl-2*H*-tetrazolium bromide) cell proliferation assay. A stock solution of MTT (cat. no. M2128, Sigma-Aldrich, Rehovot, Israel) was prepared by dissolving 5 mg of MTT in 1.0 mL of DDW. Following incubation of the cells for 24 h at 37 °C with GalSph or GalCer aggregates, the cells were washed with Dulbecco phosphate buffer saline (DPBS) and 10 μL of MTT reagent (0.5 mg/mL) was added to 90 μL of the cell culture medium followed by additional 4 h incubation at 37 °C. Next, the medium containing MTT reagent was discarded by a micropipette, and 100 μL of DMSO was added to dissolve the formazan crystals. After 30 min of incubation at RT in the dark, color intensity was measured using a microplate reader at 570 and 630 nm as reference. Results are presented as the mean and the standard error of the mean. Each experiment was repeated at least three times.

### Quantification of Apoptosis Using Annexin V-FITC in SH-SY5Y Cells

SH-SY5Y neuroblastoma cells were grown to a density of 5 × 10^4^ cells/mL in 24-well plates in DMEM/F12 (1:1) supplemented with 10% FBS and allowed to adhere overnight at 37 °C. The treatment solutions containing aggregates of GalCer (100 μM and 200 μM) or GalSph (10 μM, 25 μM, and 50 μM) were prepared as described for the cell viability assay. Cells were supplemented with the solutions followed by 24 h incubation at 37 °C. Control cells were incubated also for 24 h without the addition of GalCer and GalSph aggregates into the culture medium. A medium containing 1% DMSO was used as a vehicle. Apoptosis was monitored using an annexin V-FITC MEBCYTO Apoptosis Kit (cat. No. 4700, MBL International, MA, USA) according to the manufacturer’s instructions. Briefly, adherent cells were trypsinized, detached, and combined with floating cells from the incubated growth medium. Cells were centrifuged at 1000 rpm for 3 min and washed once with PBS. Pelleted cells were resuspended in 85 μL of annexin binding buffer. Subsequently, cells were incubated with 10 μL of annexin V-fluorescein isothiocyanate (FITC) and 5 μL of propidium iodide (PI) for 15 min in the dark. After incubation, cells were resuspended in 400 μL of binding buffer and analyzed by flow cytometry using a single laser emitting excitation light at 488 nm. Data from at least 1 × 10^4^ cells were acquired using the S100EXi flow cytometer (Stratedigm, Inc., San Jose, CA, USA) with Cell CapTure software v4.1 (Stratedigm, Inc.), and the percentages of live, apoptotic, and necrotic cells were evaluated using FlowJo v10 (FlowJo, Ashland, OR, USA). FITC log *H* and PI log *H* were expressed on *X* and *Y* axes, respectively. A quadrant was placed in a way that contained more than 95% of live cells in the control sample. The presented results were derived from three independent experiments performed in triplicate.

### Measurement of Mitochondrial Membrane Potential in SH-SY5Y Cells

Mitochondrial membrane potential (MMP) was measured using a lipophilic cationic dye 5,5,6,6-tetrachloro-1,1,3,3-tetraethylbenzimidazolylcarbocyanine iodide (JC-1, cat. no. 420200, Sigma-Aldrich, Rehovot, Israel). The treatment solutions containing aggregates of GalCer and GalSph were prepared as described above for the MTT assay. Briefly, 2 μM of JC-1 dye (from 200 μM stock solution) was added to the cells and they were maintained for 25 min at 37 °C. After incubation, the cells were washed with PBS once and resuspended in 200 μL of PBS at 37 °C. The cell samples were then subjected to a S100EXi flow cytometer (Stratedigm, Inc., San Jose, CA, USA) with CellCapTure software v4.1 (Stratedigm, Inc.) and analyzed for red/green fluorescence intensity using the FlowJo v10 (FlowJo, Ashland, OR, USA).

### In-Cell Raman Microspectroscopy for Determination of Cytochrome *b* and *c* Levels in SH-SY5Y Cells

SH-SY5Y cells were seeded on μ-dishes with a glass bottom (35 mm, high, cat. no. 81158 ibidi GmbH, Gräfelfing, Germany) in DMEM/F12 (2 mL of medium per dish). The cells were supplemented with preformed fibrils of GalCer (200 μM) or GalSph (25 μM) in the culture medium for 24 h. Before recording the Raman spectra, the medium was discarded and the cells were washed twice in PBS solution. The Raman measurements were performed at RT within 40 min per dish. A WITec alpha 300R inverted confocal Raman microspectrometer (WITec, Ulm, Germany) equipped with a 532 nm laser was used to record the spectra of the cells with objective Nikon CFI Plan Fluor 60×/0.85. The laser power per registration spot was 2.5 mW; the acquisition time was 30 s, 2 accumulations. The conditions were adjusted to avoid the photobleaching of cytochromes. Raman spectra were recorded from the cytoplasm region around the nucleus of at least 30 living cells per experimental group, distributed across 3 independent repeats. The baseline was subtracted by fitting a straight line under the peaks of interest using a custom Python script. The wavenumber values as anchor points to construct the baseline were chosen after the processing of around 50 spectra of cells. After the baseline subtraction, the intensities of the peaks of interest were defined and ratios of Raman intensities were calculated.

### Induction and Inhibition of Sphingolipid Aggregation in SH-SY5Y Cells

Intracellular accumulation of GalCer and GalSph was induced by treating the cells with a potent GALC enzyme inhibitor azo-galacto-fagomine (AGF).^53^ AGF was dissolved in DDW to prepare a 1 mM stock solution. Cells were seeded at a density of 1 × 10^5^ μ-dishes with a glass bottom (35 mm, high, cat. no. 81158 ibidi GmbH, Gräfelfing, Germany). Cells were incubated with 5 μM AGF for 5 consecutive days in DMEM/F12 culture medium. GalCer aggregates were visualized by immunofluorescence using anti-GalCer antibody under confocal microscopy (Leica TCSSP 8, Wetzlar, Germany).

For inhibition of sphingolipid aggregation, cells were treated with 5 μM of AGF for 5 consecutive days followed by supplementation of different concentrations of naphthoquinone-tryptophan hybrid (NQTrp, 5 μM, 10 μM, 20 μM, 50 μM, and 100 μM) for additional 48 h. Reduction of GalCer puncta compared to cells nontreated with NQTrp was evaluated by confocal imaging. Fluorescence intensity was quantified by using ImageJ.

### Immunofluorescence Detection of GalCer Aggregates in SH-SY5Y Cells

The intracellular GalCer aggregation was investigated by subjecting AGF treated SH-SY5Y cells, with or without NQTrp, as described above. The cells were next washed three times in DPBS, 5 min each, fixed with 4% PFA (in DPBS) for 10 min, washed (3 × 10 min) in PBS, and then permeabilized for 10 min in 0.1% Triton X-100 in DPBS. The cells were blocked with a blocking solution containing 0.1% Tween 20 and 3% bovine serum albumin (BSA) solution in DPBS for 2 h. Then they were incubated with primary rabbit polyclonal anti-GalCer antibodies (cat. no. RAS_0030, Hamburg, Glycobiotech, Germany) overnight at 4 °C (diluted 1:600 into 3% PBS with 0.1% Tween-20 (BSA-PBST)). On the next day, the cells were washed (3 × 15 min) in DPBS and incubated for 1 h at RT with Cy3-conjugated goat antirabbit secondary antibody (diluted 1:800 into 3% BSA-PBST) (Cat. no. 111-165-003, Jackson Immuno Research, PA, USA). Cells were imaged using a Leica TCS SP8 laser (DAPI: *E*_*x*_ = 405 nm, *E*_m_ = 418–480 nm; Cy-3: *E*_*x*_ = 561 nm, *E*_m_ = 571–640 nm) confocal microscope with 63 ×1.4 NA oil objectives (Leica Microsystems, Wetzlar, Germany) and 1 pinhole airy. Fluorescence intensity was quantified using ImageJ.

### Statistical Analysis

GraphPad Prism version 8 (GraphPad Software Inc., La Jolla, CA, USA) was used for statistical analysis. The data shown in this study are mean ± standard error of the mean (SEM). All results are expressed as the mean ± SD of three independent experiments. The measurements were statistically analyzed using analysis of variance (ANOVA) followed by a Tukey multiple comparison test. The level of significance was set at *p* < 0.05.

## Supplementary Material



## Notes

End point ThS intensity of GalCer and GalSph at PBS (pH 7.4) and AB (pH 4.5); well view of turbidity assay of GalCer and GalSph at different concentrations; TEM images of GalCer and GalSph fibril formation at different time points; TEM images of GalCer and GalSph fibrils after 30 days and 60 days incubation period; TEM images of GalCer and GalSph fibrils incubated at different temperatures; TEM images of GalCer and GalSph fibril formation in DMEM/F12 medium; MTT assay of monomeric GalCer and GalSph to SH-SY5Y cells; and GalCer aggregation in SH-SY5Y cells after treatment with different concentrations of GALC inhibitor (PDF)

## Abbreviations

AGF: azo-galacto-fagomine
ANS: 8-anilinonaphthalene-1-sulfonic acid
CR: Congo red
DDW: double-distilled or deionized water
DMSO: dimethyl sulfoxide
DPBS: Dulbecco phosphate buffer saline
EGCG: epigallocatechin-3-gallate
FTIR: Fourier-transform infrared spectroscopy
GALC: galactosylceramidase
GalCer: galactosylceramide
GalSph: galactosylsphingosine
GCase: glucosylcerebrosidase
GD: Gaucher disease
GLD: globoid cell leukodystrophy
GlcCer: glucosylceramide
JC-1: 5,5,6,6′-tetrachloro-1,1′,3,3′-tetraethylbenzimidazolylcarbocyanine iodide
KD: Krabbe disease
LSDs: lysosomal storage diseases
MMP: mitochondrial membrane potential
MTT: (3-(4,5-dimethyl-2-thiazolyl)-2,5-diphenyl-2*H*-tetrazolium bromide)
NQTrp: naphthoquinone-tryptophan hybrid
PTA: phosphotungstic acid
PXRD: powder X-ray diffraction
SAXS: small-angle X-ray scattering
TEM: transmission electron microscopy
ThS: Thioflavin S
