# Correction to “Self-Assembly of Accumulated Sphingolipids into Cytotoxic Fibrils in Globoid Cell Leukodystrophy and Their Inhibition by Small Molecules in Vitro”

**DOI:** 10.1021/acsnano.5c14040

**Published:** 2025-09-10

**Authors:** Sourav Kumar, Evelina Nikelshparg, Jana Pilátová, Ashim Paul, Vijay Kumar, Gil Koren, Dana Laor Bar-Yosef, Roy Beck, Henrik H. Jensen, Daniel Segal

Dr. Laor Bar-Yosef’s name was inadvertently not included in the original version of the manuscript, and I am sorry for that.

Dr. Laor Bar-Yosef was significantly involved in the introduction and use of the Raman microspectroscopy part of this project. She was a major contributor to the novel conceptual use of this technology for detecting and monitoring sphingolipids in vitro and in cells. As expected from her deep involvement, Dr. Laor Bar-Yosef took part in analyzing the corresponding results.

The revised correct order of authors, approved by all coauthors, should be Sourav Kumar, Evelina Nikelshparg, Jana Pilátová, Ashim Paul, Vijay Kumar, Gil Koren, Dana Laor Bar-Yosef, Roy Beck, Henrik H. Jensen and Daniel Segal.

This correction does not affect the results or conclusions of the paper.

