# Supplementary material for: Self-Assembly of Accumulated Sphingolipids into Cytotoxic Fibrils in Globoid Cell Leukodystrophy and Their Inhibition by Small Molecules In Vitro
Source: ACS Nano. 2025 Jul 2;19(27):25180–203. doi: 10.1021/acsnano.5c05498 (PMC12269364; doi:10.1021/acsnano.5c05498)
Supplement: Supplementary file 1 [file nn5c05498_si_001.pdf]

Supporting Information

Self-assembly of Accumulated Sphingolipids into Cytotoxic Fibrils in Globoid Cell Leukodystrophy and their Inhibition by Small Molecules *in vitro*

*Sourav Kumar[1], Evelina Nikelshparg[2], Jana Pilátová[1,3,4], Ashim Paul[5], Vijay Kumar[1], Gil Koren[6], Roy Beck[6,8], Henrik H. Jensen[7] and Daniel Segal[*1,8]*

[1]Shmunis School of Biomedicine and Cancer Research, George Wise Faculty of Life Sciences, Tel Aviv University, Tel Aviv 6997801, Israel

[2]Department of Life Sciences, Ben-Gurion University of the Negev, Beer Sheva 8855630, Israel.

[3]Institute of Physics, Faculty of Mathematics and Physics, Charles University, Prague 2 121 16, Czech Republic

[4]Molecular Foundry, Lawrence Berkeley National Laboratory, Berkeley 94720, California, USA

[5]Structural Biology & Bioinformatics Division, Indian Institute of Chemical Biology, Raja S. C. Mullick Road, Jadavpur, Kolkata – 700032, India

[6]The Raymond & Beverly Sackler School of Physics and Astronomy, The Center for Nanoscience and Nanotechnology, and the Center for Physics and Chemistry of Living Systems, Tel Aviv University, Tel Aviv 6997801, Israel.

[7]Department of Chemistry, Aarhus University, 8000 Aarhus C, Denmark

[8]Sagol School of Neuroscience, Tel Aviv University, Tel Aviv 6997801, Israel.

[*]Corresponding Author

Daniel Segal

Shmunis School of Biomedicine and Cancer Research, George Wise Faculty of Life Sciences, Tel Aviv University, Tel Aviv 6997801, Israel; Sagol Interdisciplinary School of Neuroscience, Tel Aviv University, Tel Aviv 6997801, Israel; Phone: +972-3-640-9835; Fax: +972-3-640-9407; orcid.org/0000-0002-6777-505X; Email: dsegal@post.tau.ac.il, dsegal@tauex.tau.ac.il

Key words. Globoid Cell Leukodystrophy, Sphingolipids, Self-assembly, Galactosylceramide, Galactosylsphingosine, Small molecule inhibitors, Apoptosis.

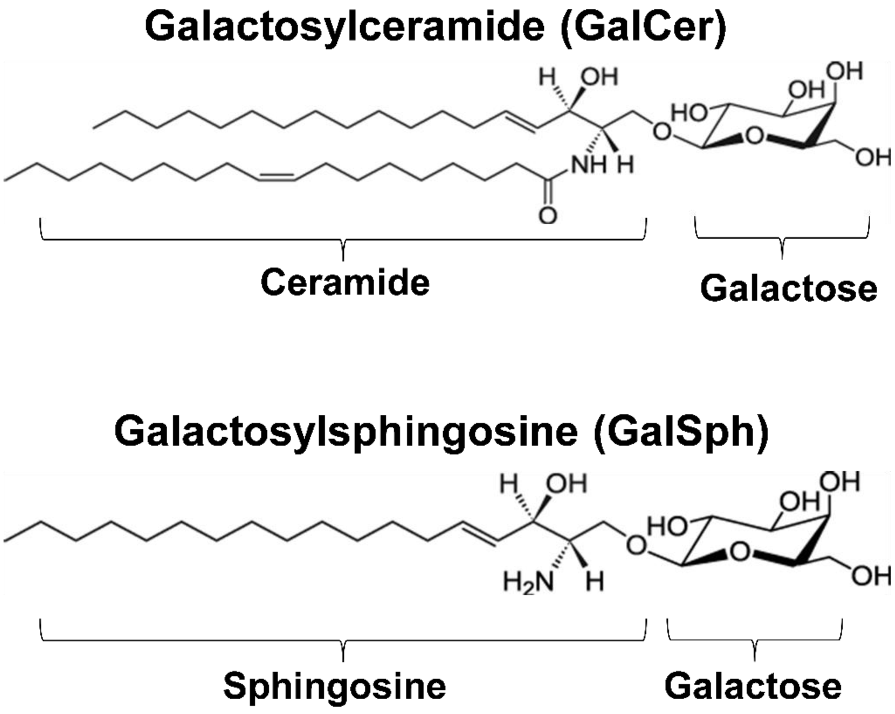

Figure S1. Chemical structure of galactosylceramide (GalCer) and galactosylsphingosine / psychosine (GalSph). D-galactosyl-β-1,1′-N-oleoyl-D-erythro-sphingosine (GalCer) or C18:1 galactosyl(β) ceramide (d18:1/18:1(9Z)), empirical formula: $C_{42}H_{79}NO_8$, MW: 726.08 and D-galactosyl-β1-1′-D-erythro-sphingosine (GalSph) or galactosyl(β) sphingosine (d18:1), empirical formula: $C_{24}H_{47}NO_7$, MW: 461.63 were used in the present study.

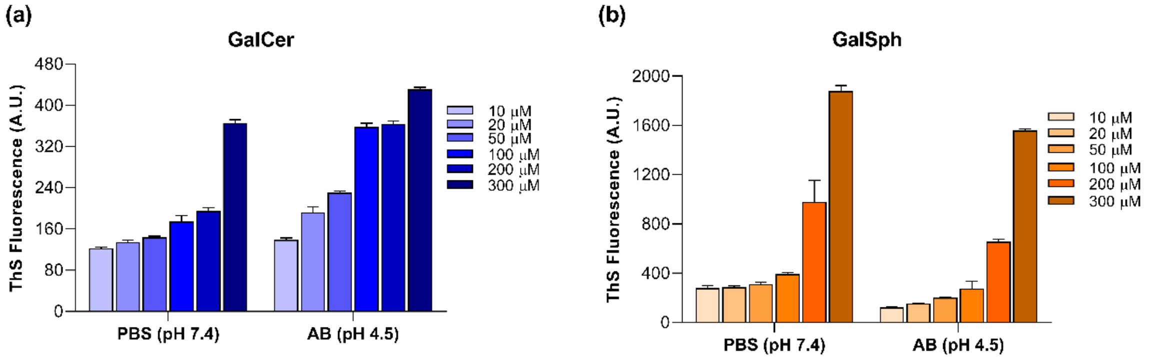

Figure S2. End point ThS intensity of (a) GalCer and (b) GalSph at PBS (pH 7.4) and AB (pH 4.5). Bar diagram represents the end-point ThT fluorescence for the aggregation of different concentrations (10-300 µM) of GalCer and GalSph. Experiments were repeated at least three times with similar observations. Data are mean ± SEM.

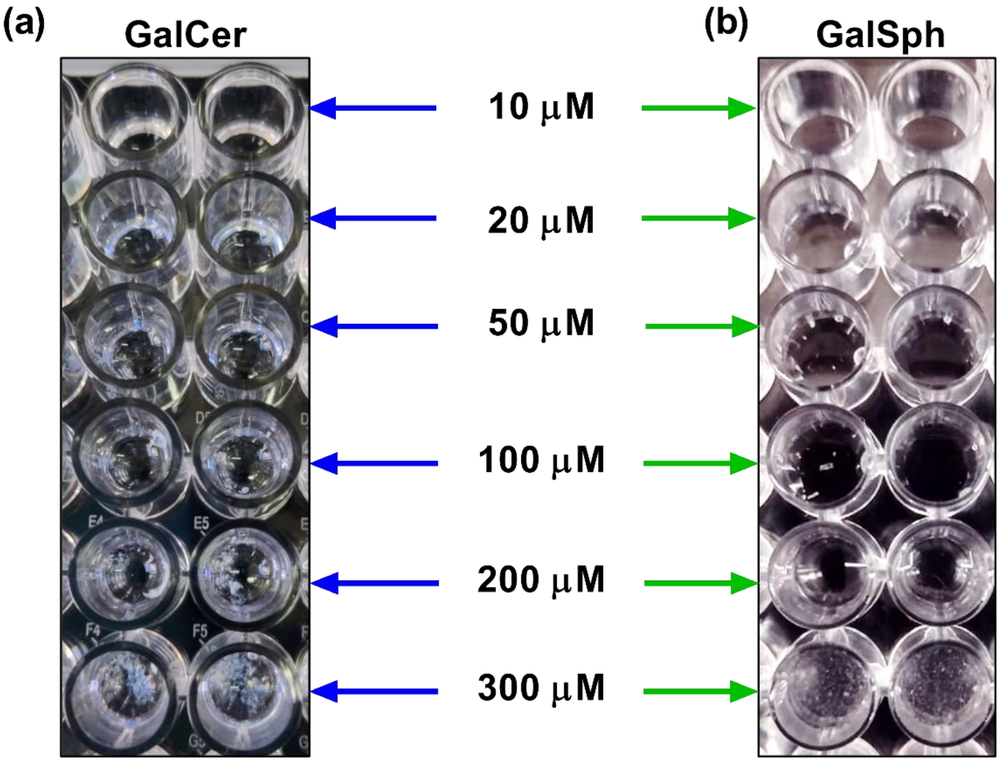

Figure S3. Well view of turbidity assay of (a) GalCer and (b) GalSph at different concentrations. Images were taken after 24 h of incubation of both sphingolipids at different concentrations (10-300 μM) in PBS pH 7.4 at 37 °C and showing white color aggregates inside the wells.

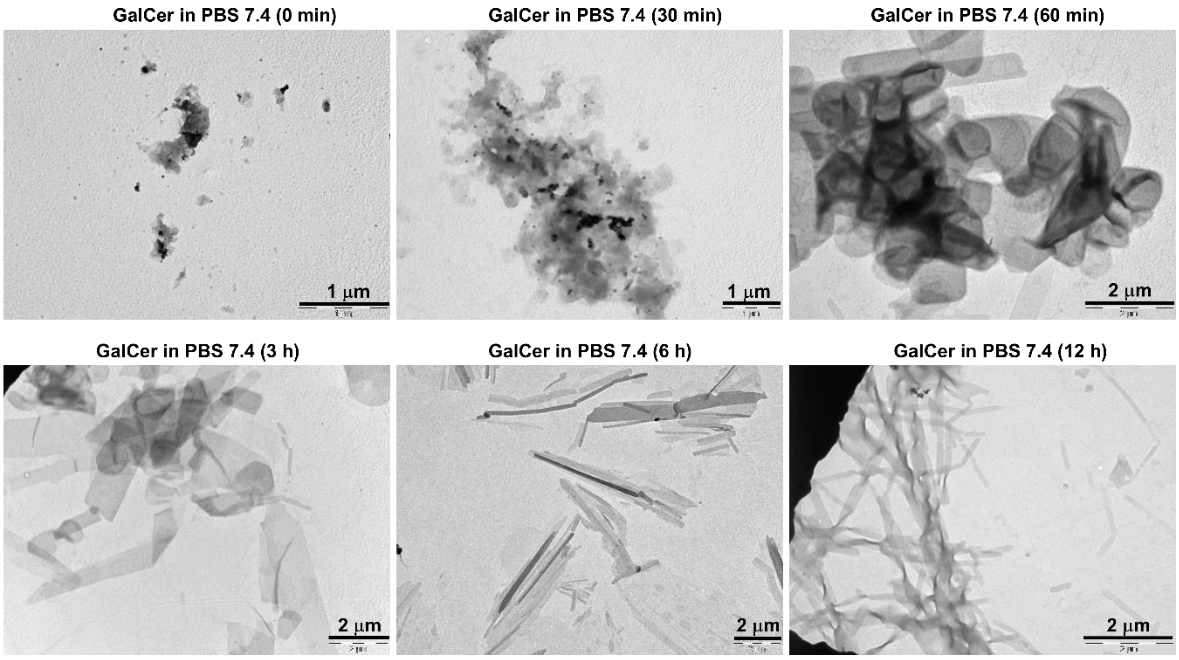

Figure S4. TEM images of GalCer (100 μM) in PBS pH 7.4. Images were taken at different time points after dilution in PBS from the original stock of GalCer (10 mM in DMSO).

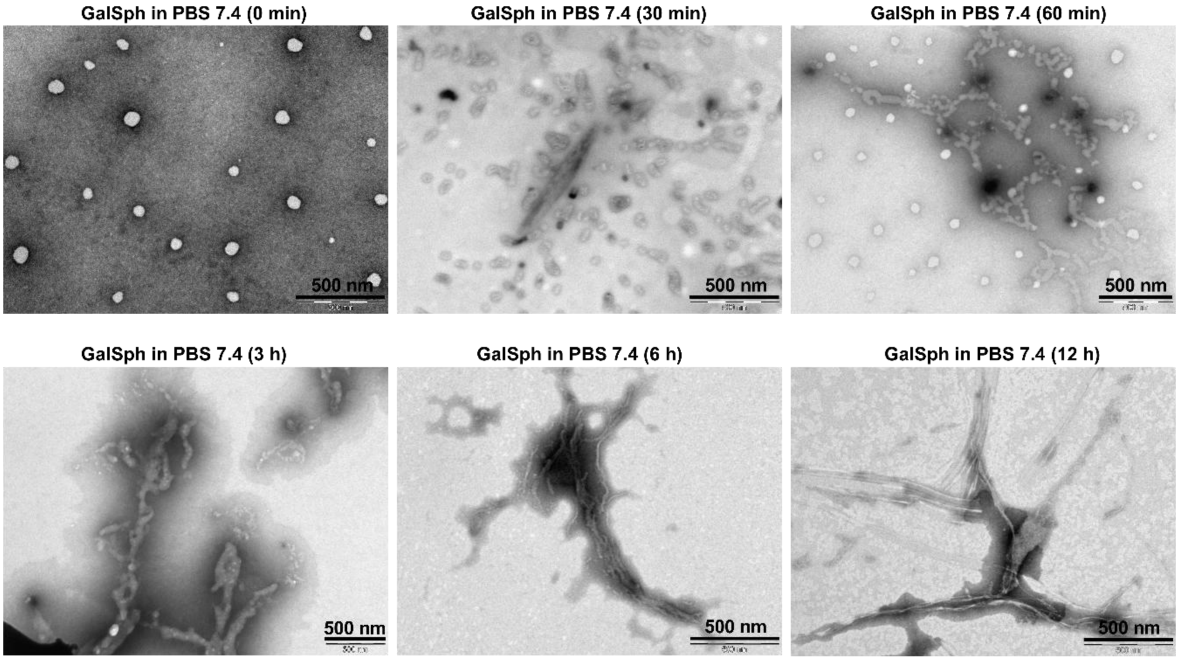

Figure S5. TEM images of GalSph (100 μM) in PBS pH 7.4. Images were taken at different time points after dilution in PBS from the original stock of GalSph (10 mM in DMSO).

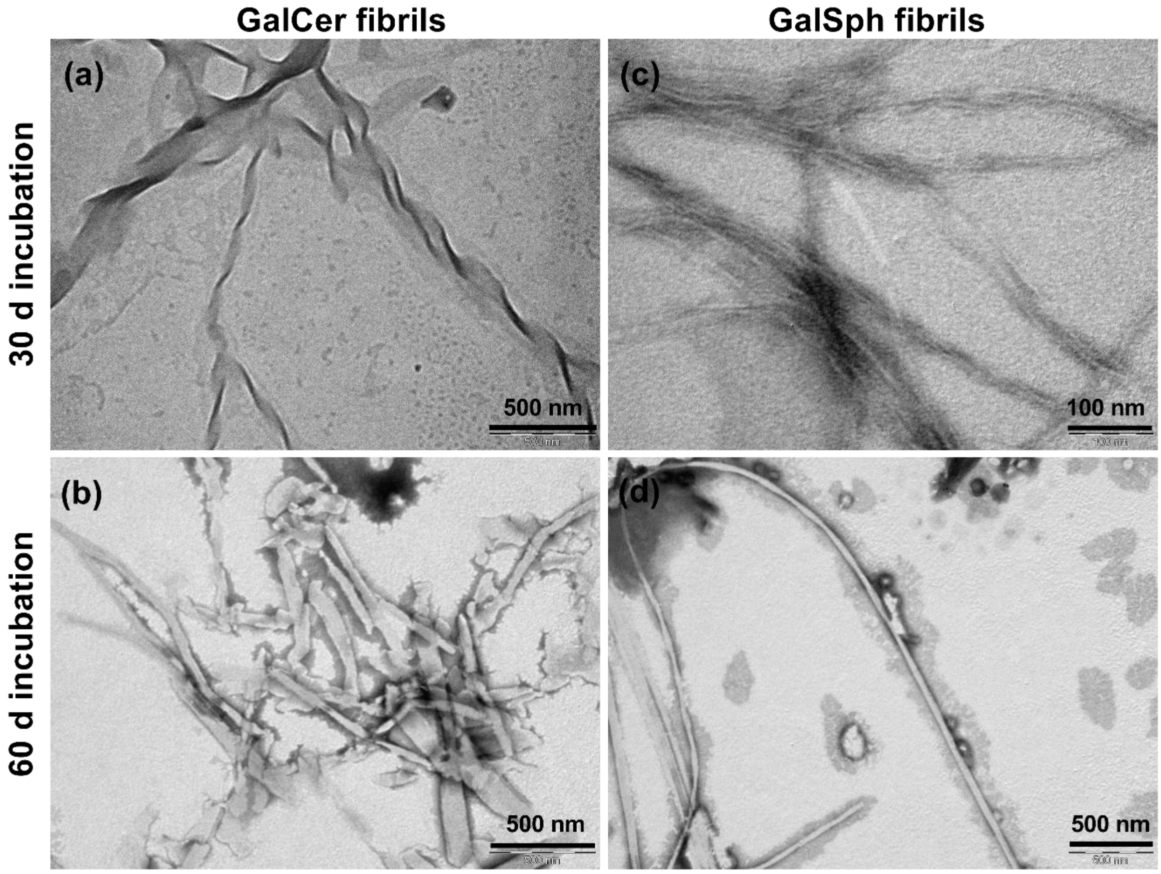

Figure S6: TEM images of GalCer (a,b) and GalSph (c, d) in PBS pH 7.4. Images were taken after 30 days and 60 days of incubation of GalCer and GalSph at 37 °C.

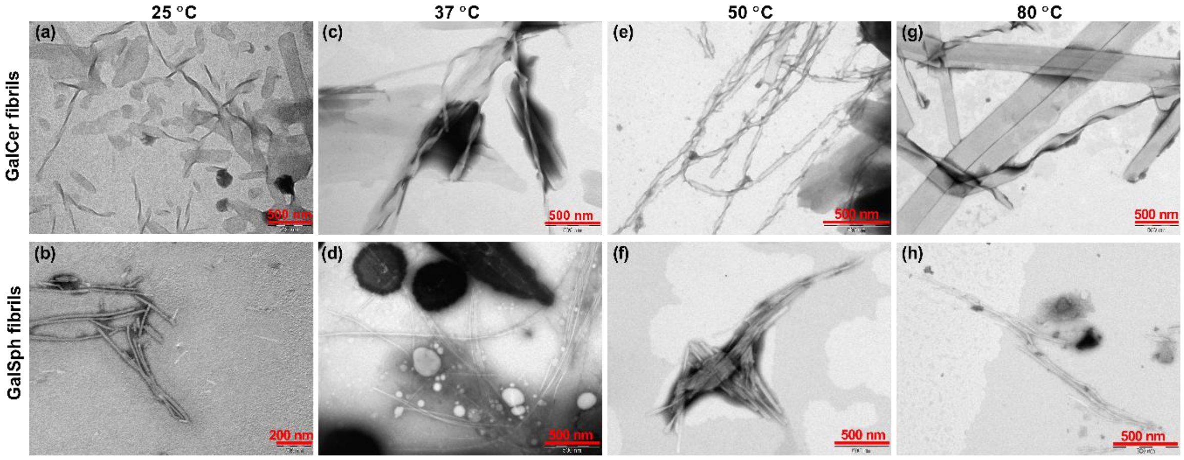

Figure S7: TEM images of GalCer (100 µM) and GalSph (100 µM) fibrils incubated at different temperatures. GalCer (100 µM) and GalSph (100 µM) monomers were first incubated for 24 h to generate preformed fibrils (PFFs). GalCer and GalSph PFFs were further incubated for 24 h at different temperatures: (a, b) 25 °C, (c,d) 37°C, (e, f) 50°C, (g, h) 80 °C to check the stability of the fibrils.

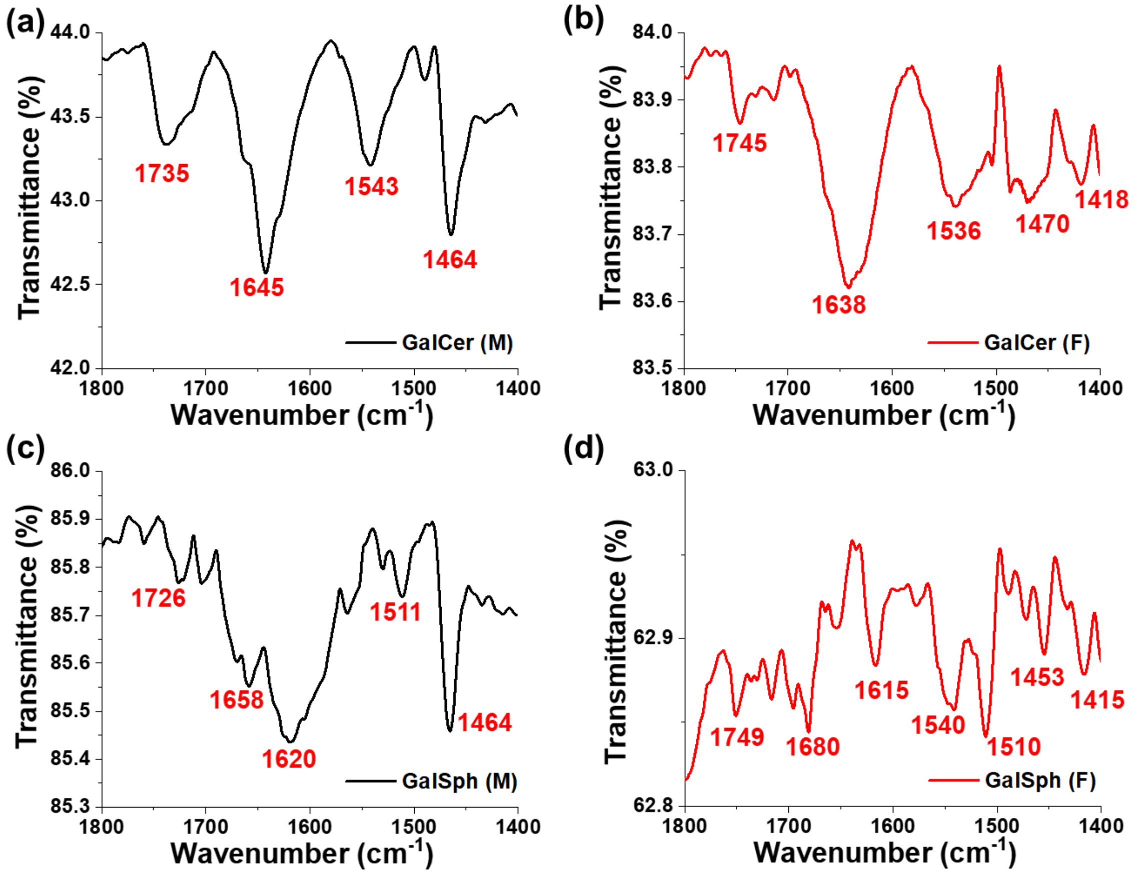

Figure S8: Expanded FTIR spectra of GalCer and GalSph. FTIR spectra of the expanded region of Figure 4a, b (1400-1800 cm^{-1})) of the GalCer monomers (a), and fibrils (b) and Figure 4c, d (1400-1800 cm^{-1})) of the GalSph monomers (c), and fibrils (d).

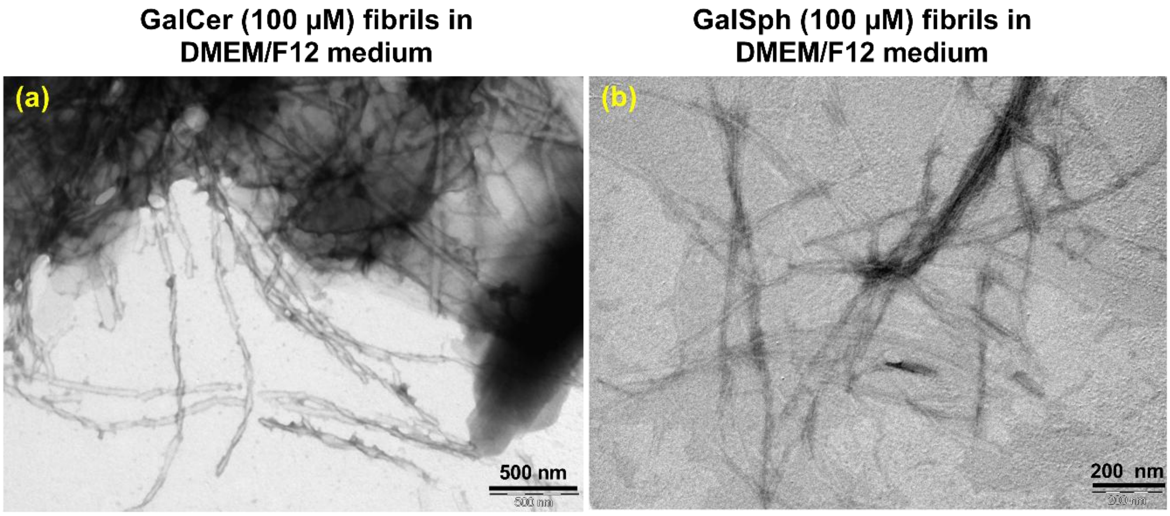

Figure S9: TEM images of GalCer and GalSph in DMEM/F12 medium. Samples were taken after 24 h incubation of GalCer (100 µM) and GalSph (100 µM) in the culture media at 37 °C, and TEM imaging was performed.

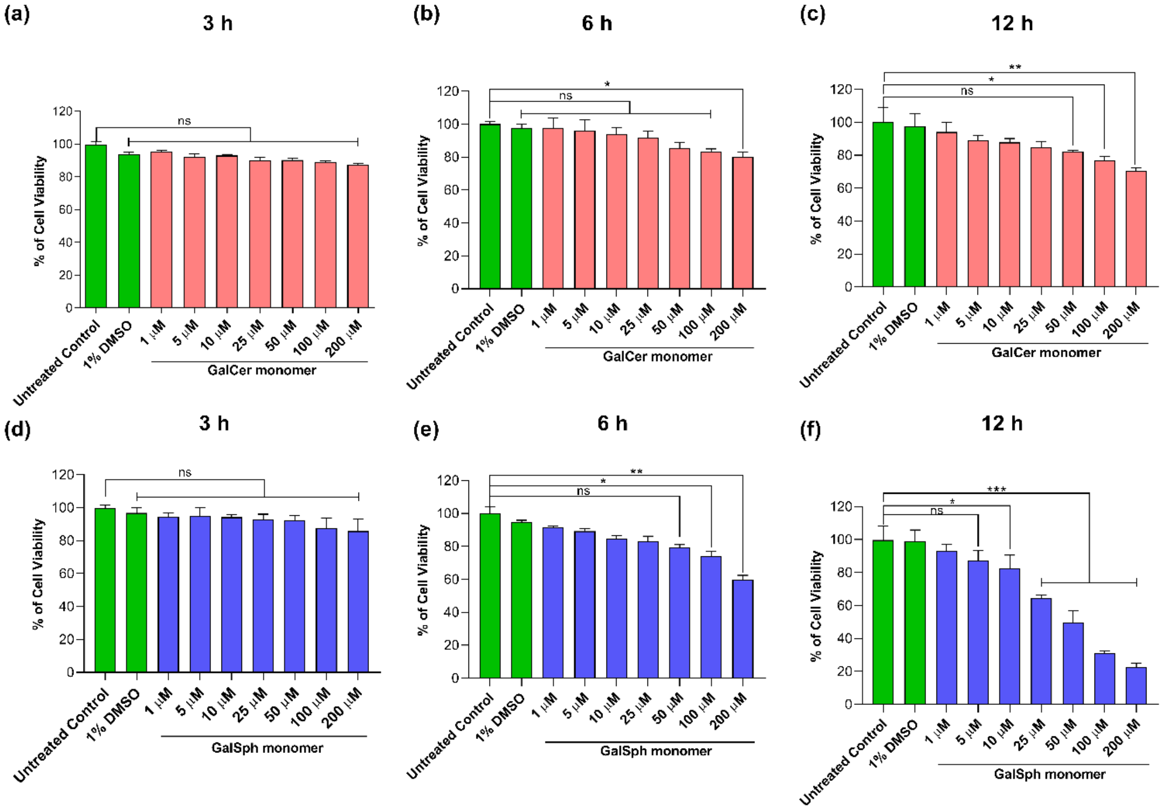

Figure S10. Cytotoxicity of GalCer and GalSph to SH-SY5Y cells. Cells were incubated with monomeric GalCer for (a) 3 h, (b) 6 h, (c) 12 h, and GalSph for (d) 3 h, (e) 6 h, (f) 12 h, and cell viability was measured by MTT assay. Values are represented as mean ± SEM, $n = 6$. Significance was analyzed using one-way ANOVA followed by Tukey multiple comparison test, *p <0.05, **p <0.01, ***p <0.001.

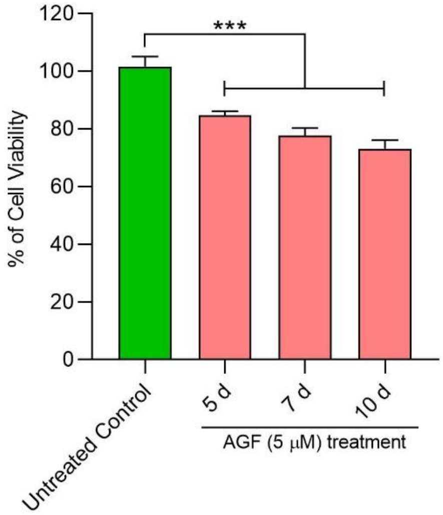

Figure S11: Cytotoxic effects of endogenous sphingolipids assessed by MTT cell viability assay. SHSY-5Y cells were treated with 5 μM GALC inhibitor (AGF) up to 10 d and cell viability was determined on day 5, 7 and 10 using the MTT assay. The data are represented as percentage cell viability. Each bar represented as mean ± SEM, $n = 6$. Statistical significance was analyzed using one-way ANOVA followed by Tukey multiple comparison post-hoc test, ***p <0.001 vs. untreated control.

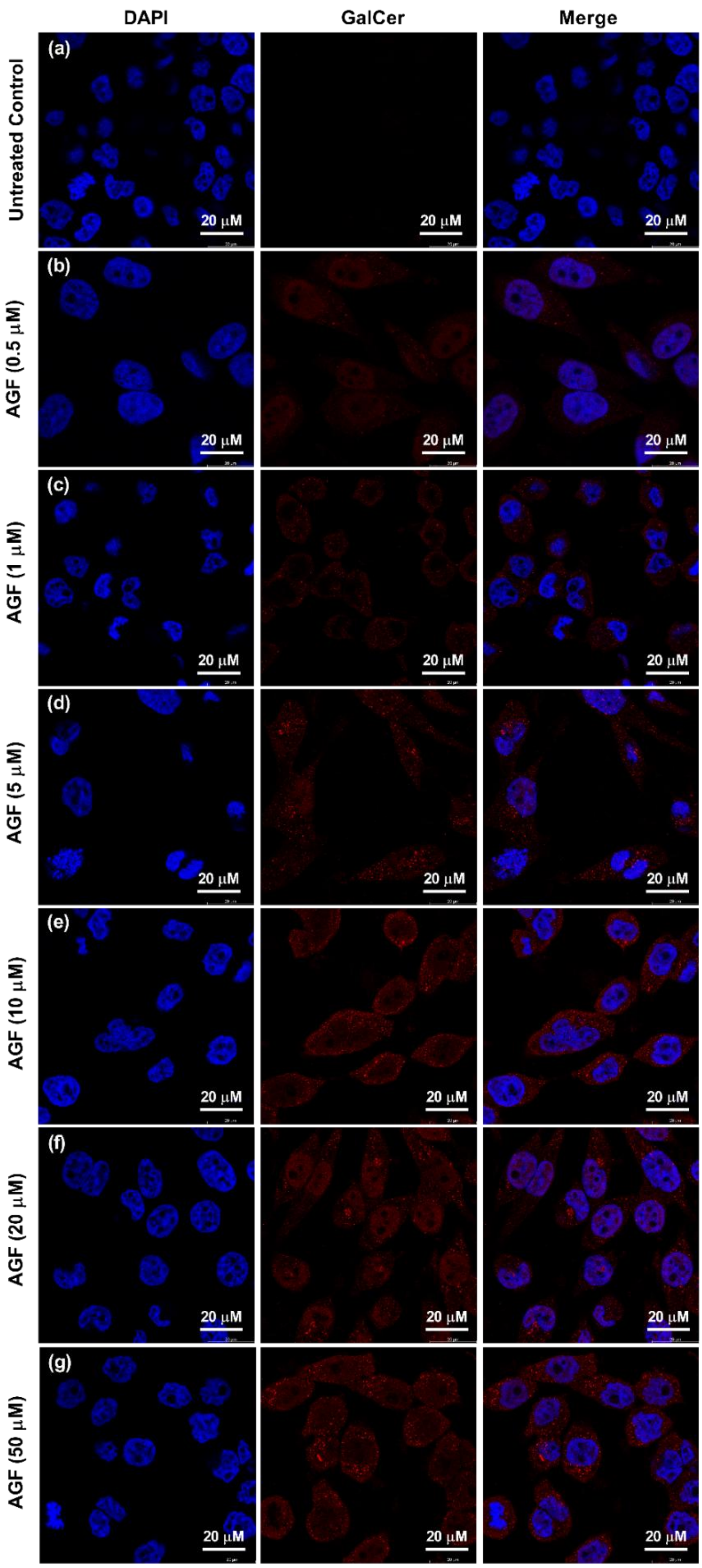

Figure S12. GalCer aggregation in SH-SY5Y cells after treatment with different concentrations of GALC inhibitor (AGF). Cells were incubated and maintained with different concentrations (0.5-50 μM) of AGF for 5 consecutive days. GalCer aggregation was detected by immunofluorescence staining against GalCer with specific antibodies. AGF untreated cells serve as a control. Images indicate (a) untreated control without GalCer puncta and (b-g) GalCer puncta in AGF-treated cells.
